# Advances in Urinary Liquid Biopsy: A Review of Emerging Sensing Platforms for Early Cancer Screening and Diagnosis

**DOI:** 10.3390/s26196258

**Published:** 2026-10-02

**Authors:** Eleni Lamprou, Dimitrios Papakyriakou, Despina P. Kalogianni

**Affiliations:** Analytical Chemistry & Nanotechnology Group, Department of Chemistry, University of Patras, GR-26504 Patras, Greece

**Keywords:** liquid biopsy, urine, prostate cancer, bladder cancer, nanotechnology, sensor, biosensor, sensors artificial intelligence, machine learning, deep learning

## Abstract

Early screening of many types of cancers remains limited by the invasiveness of gold-standard tissue biopsies and the poor specificity of traditional clinical biomarkers like prostate-specific antigen (PSA). Non-invasive liquid biopsies are the emerging alternatives. Urine is one of the preferred body fluids for liquid biopsy as it offers a rich, easily accessible and large-volume source of cancer-specific metabolites, proteins, nucleic acids, extracellular vesicles, and cancer cells. This review reports the recent advancements in urinary cancer (bio)sensing technologies, including mainly optical and electrochemical platforms. To overcome the low specificity and detectability of the existing systems, novel nanomaterials, such as metal–organic frameworks, carbon dots, metal nanoparticles, and molecularly imprinted polymers, that offer increased surface area, cost-effectiveness and enzyme-free platforms, as well as accelerated electron transfer, were introduced to provide lower detection limits to the femtomolar and attomolar levels. Additionally, the integration of machine learning and artificial intelligence for biochemical “fingerprint” analysis enhanced diagnostic accuracy. Finally, we discuss the gap between novel sensing systems and robust diagnostic devices for decentralized point-of-care testing (POCT) and personalized medicine in clinical routine.

## 1. Introduction

Diagnostic methods for some types of cancer, such as prostate cancer (PCa), are still limited in terms of invasiveness and specificity. PCa and other types constitute a major global health issue. Early diagnosis is extremely important to improve survival rates. In PCa, prostate-specific antigen (PSA) testing has limited specificity and may lead to false-positive results, unnecessary biopsies and overdiagnosis [1]. Pathologic biopsy and cystoscopy are currently the main methods for the diagnosis of bladder cancer, but they are invasive with high costs and low sensitivity. In addition, the sensitivity of conventional white-light cystoscopy for bladder tumor detection has been reported to range from 62% to 84% with lower sensitivity for small or flat lesions [2]. Similarly, carbohydrate antigen 19-9 (CA19-9), which is widely used in pancreatic cancer, has reported sensitivities of 79–95% and specificities of 82–91%, while elevated levels may also occur in benign conditions [3]. These limitations support the development of non-invasive methods that target urine biomarkers for early screening and diagnosis. Analytical sensitivity or a low limit of detection alone does not establish diagnostic efficacy. Clinical evaluation should also consider comparison with an appropriate reference method and diagnostic measures such as sensitivity, specificity, AUC and, where applicable, positive and negative predictive values in clinically relevant patient populations [1]. Liquid biopsy, as defined by the International Society of Liquid Biopsy, involves the analysis of various components such as cells, nucleic acids, proteins, metabolites, and extracellular vesicles, obtained from several bodily fluids collected through minimally invasive methods [4,5]. However, these analyte classes are not biologically equivalent and can provide different types of biological information. Urinary nucleic acids, proteins, extracellular vesicles and metabolites originate through different biological processes and therefore differ in their relationship to tumor biology and in their interpretation as cancer. Thus, although these analytes can all contribute to urinary liquid-biopsy approaches, they should not be considered biologically interchangeable biomarkers [6].

Established clinical biomarkers, e.g., prostate-specific antigen (PSA), have clinical limitations due to low specificity, giving false-positive results. Therefore, new biomarkers with advanced specificity in non-invasive diagnosis are in urgent demand. Urine biomarkers are the ideal candidates, as urine is the most easily accessible body fluid of large volume, whereas it comprises a rich source of biomarkers and various components that provide valuable information for health status. Urine is also valuable for metabolomics-based biomarker discovery, as it contains a large number of endogenous metabolites [7,8,9,10]. Urinary biomarker panels including DNA methylation, microRNAs, peptides and metabolites have also been explored for non-invasive cancer detection [11]. In addition, abnormal protein expressions and altered metabolite secretions are also considered as candidate biomarkers for liquid biopsy in urine [12]. However, the use of urine biomarkers in clinical routine diagnosis is still challenging due to the extremely complex and variable matrix, as well as the very low concentration of the analytes [7,8,9,10]. Metabolites from cancer cells are secreted into urine either through systemic circulation and filtration by the kidneys, or for cancers of the urinary system via direct secretion from tumor cells into the urinary tract [13,14].

Several techniques have been used for the detection of cancer biomarkers including UV-visible spectroscopy, fluorescence, liquid chromatography-mass spectrometry (LC-MS), high-performance liquid chromatography (HPLC), mass spectrometry (MS) and enzyme-linked immunosorbent assay (ELISA), in combination with imaging technologies, and histopathological evaluation. However, these techniques are time-consuming, require special and costly equipment and highly trained personnel. Often, a sample pre-treatment step is also needed, while ELISA suffers from poor selectivity. As the demand is toward point-of-care testing (POCT) and personalized medicine, novel (bio)sensors of low-cost that provide fast, accurate and specific analysis are currently the state-of-the-art in diagnostics. Advancements in nanotechnology have offered elevated analytical performance of the developed biosensors. New optical reporters provided enhanced optical properties, high quantum yield, photostability, and low toxicity. Electroanalytical techniques, in particular, are promising diagnostic tools for POCT due to their great simplicity, low cost, rapid analysis and excellent detectability. These techniques include cyclic voltammetry (CV), differential pulse voltammetry (DPV), and electrochemical impedance spectroscopy (EIS). The use of novel nanomaterials and nanostructures, such as metal–organic frameworks (MOFs), other nanoporous materials, and metal nanoparticles, enhanced electron transfer and increased the surface area available for the binding of biorecognition molecules, improving the overall analytical performance and especially the detectability of the sensors. Moreover, these improvements could be further enhanced by combining nanostructures with complementary properties that produce a synergetic effect [8,10]. In addition, in some types of cancer the disease exhibits high heterogeneity and single-biomarker detection fails to provide sensitive and specific diagnosis. Multiplex analytical devices in combination with sample enrichment approaches are the key to a more accurate and earlier diagnosis [15]. Furthermore, dual-mode analysis sensors have been constructed by integrating two sensing technologies into a single platform to allow for more accurate analysis and reduce false-positive results [16].

Attempts have been made to construct enzyme-free sensors, because enzyme-based sensors are vulnerable to interferences from complex matrices leading to false results, and increase the cost, the number of steps or the total time of the analysis. Novel nanomaterials, like metal or bimetallic nanoparticles, MOFs, and carbon-based nanomaterials, have emerged here as nanozymes, introducing enzyme-mimetic behavior like oxidase and peroxidase activity with the advantages of low cost, easy fabrication, high stability and specificity even in complex matrices like urine [8,17]. Moreover, to lower the cost of fabrication and increase the specificity of the sensors, aptamers have been introduced as alternatives to antibodies. Aptamers are synthetic single-stranded DNA (ssDNA) sequences that exhibit excellent affinity for specific targets. To facilitate measurements with enhanced detectability, aptamers are combined with various nanomaterials [18]. Also, molecularly imprinted polymers (MIPs) are synthetic materials constructed with artificial molecular recognition sites that mimic natural biorecognition molecules, such as antibodies and enzymes and are important alternatives to enzymes. They are also cost-effective, robust and have outstanding anti-interference performance, therefore providing specific detection of urine analytes without tedious sample pretreatment. The integration of other nanomaterials to MIPs, such as fluorescent carbon dots, greatly enabled real-time monitoring and quantification ability [10,19].

Novel urine biomarkers include prostate Cancer Antigen 3 (PCA3) ─ a long noncoding RNA (lncRNA), prostate-specific membrane antigen (PSMA), sarcosine (SAR) that is an amino acid derivative elevated in PCa tissue compared to normal prostate tissue, spermine that plays a role in secretory functions, all for early-stage PCa detection, carcinoembryonic antigen (CEA), leucine and human chorionic gonadotropin (hCG) for various types of cancer, nuclear matrix protein 22 (NMP22) and hyaluronidase for bladder cancer, carbohydrate antigen 19–9 (CA 19–9) for pancreatic cancer, vanillylmandelic acid for detection of neuroblastomas, and various nucleic acids with more prominent the microRNAs, as well as tumor-derived extracellular vesicles like exosomes, and circulating cancer cells. In addition, exosomal miRNAs or other exosomal components derived from tumor cells provide valuable and promising non-invasive biomarkers for early diagnosis and assessment of the efficacy of the applied therapy. However, accurate detection and identification of miRNAs or other molecules within exosomes is extremely challenging due to their low abundance along with the complex and tedious extraction of exosomes and exosomal components from body fluids [20]. The biomarkers discussed in the review are presented according to their use and description in the original sensing studies, with emphasis on their role as analytical targets rather than on their stage of clinical validation.

This review reports current advancements in non-invasive cancer diagnostics that utilize emerging urine biomarkers as alternatives to tissue biopsy and traditional diagnostic techniques. Researchers are developing novel biosensors that involve colorimetric, fluorescent, chemiluminescent, surface-enhanced Raman spectroscopy (SERS)-based, surface plasmon resonance (SPR), and electrochemical sensors to detect a wide variety of markers such as metabolites, small molecules, proteins, and nucleic acids. These modern systems incorporate novel and sophisticated nanomaterials like MOFs to improve detectability and provide rapid and simple analysis, aiming for their implementation for POCT. Furthermore, the integration of artificial intelligence to biosensors enables the analysis of complex biochemical ‘fingerprints’ to increase diagnostic accuracy. All reported innovations focus on the early detection and prognosis of various types of cancer, like prostate and bladder cancer.

## 2. Emerging Sensing Platforms for Urinary Liquid Biopsy

### 2.1. Methods of Record Search and Selection

Records were identified using the Scopus database by Elsevier. The search terms used were “sensors” AND “urine” AND “cancer detection”, with the publication year from 2024 to 2026 and the document type limited to “article”. Record subjects were limited to “chemistry”, “engineering”, and “chemical engineering”. The first search was conducted on 21 April 2026, and the final search was conducted on 29 July 2026. All the articles were published in English; thus, no language limitations were encountered. Articles indexed exclusively in other databases may not have been identified. Records were assessed for eligibility by the authors to exclude reviews misrepresented as articles; works concerning analytical workflows without biosensors; works that were not intended to perform liquid biopsy in urine or did not include validation in clinical urine, spiked urine, animal urine, or artificial urine samples; and sensors that were incapable of detecting cancer at an early stage. The criteria for record exclusion were pre-defined to provide selected articles that were relevant to the scope of the present review and mitigate selection bias.

### 2.2. Colorimetric, Fluorescent and Chemiluminescent Sensors

Optical detection is one of the techniques of choice in POCT platforms, as it provides enhanced simplicity and cost-effectiveness, along with rapid analysis. Among optical methods, fluorescence-based ones provide high detectability [21]. Colorimetric methods have the advantages of enhanced simplicity and rapid analysis, but, in some cases, they lack detectability compared to other methods. To overcome these limitations, they are usually combined with enzymes, nanozymes or special nanostructures for signal enhancement [22].

#### 2.2.1. Metabolites and Small Molecules

Numerous biosensors have targeted sarcosine, a naturally occurring amino acid derivative, also known as N-methylglycine, which is a potential early prostate cancer biomarker exceeding a concentration of 3 μM [22,23]. Sarcosine has been detected in invasive prostate cancer cell lines at a concentration above that of benign prostate epithelial cells using gas chromatography-mass spectrometry, while high sarcosine concentration in urine samples of patients has been correlated with the presence of cancerous prostate cells detected using standard biopsy [24]. However, the merit of urinary sarcosine as a prostate cancer biomarker is still under investigation [25]. The fundamental principle of most sarcosine biosensors is an enzyme cascade initiated by sarcosine oxidase (SOx) that is then coupled to the oxidation of a reporter molecule. Jin et al. 2024 developed a portable and cost-effective paper-based colorimetric sensor for the simultaneous detection of glucose, uric acid, and sarcosine (SAR) in urine with an LOD of 1 μM for all targets. Glucose and uric acid detection were relevant for the diagnosis of other diseases such as diabetes and gout, respectively. The sensor’s analytical performance regarding sarcosine gave a linear range of 2–500 μM and an analysis time of 34–44 min. The recoveries were in the range of 95.4–107% and RSDs < 2.8%. Sample incubation with SOx and other target-specific oxidases catalyzed the oxidation of sarcosine, glucose and uric acid, generating H_2_O_2_. The sample was added to the sensor’s paper surface, where horseradish peroxidase (HRP) had been previously immobilized via boronate affinity. In the presence of H_2_O_2_, the reaction of HRP and 3,3′,5,5′-tetramethylbenzidine (TMB) led to the generation of a blue color. Smartphone-based detection improved ease of use and portability and enabled quantification of the color change using the Blue/Red (B/R) ratio, but the analysis was dependent on consistent lighting conditions. Paper-based sensors with smartphones as detectors have been used widely in POCT due to their simplicity and low-cost analysis, whereas smartphones account for the increased user-friendliness of POCT devices and flexibility [26]. Increased selectivity and reduced susceptibility to interferents was achieved by employing a dual colorimetric/fluorometric assay to cross-validate signals. An example is a sensor using p-phenylenediamine (PPD) and fluorescent carbon dots (CDs) as labels. SOx and HRP were encapsulated within a zirconium metal–organic framework (Zr-MOF) with peroxidase-like activity to improve cascade kinetics via substrate channeling and detectability due to a high surface area. The enzyme cascade produced oxidized PPD, which served as both a chromophore and a fluorescence quencher. The LOD reached 0.44 μM for colorimetric detection and 0.21 μM for fluorometric detection with a good linear relationship in the range of 0.5–200 μM. The total analysis time for urine samples was 51 min and the spiked urine samples recoveries for both methods were in the range of 97.3–104.2% with RSDs of 0.78–3.75% [27].

Enzyme components in sensors raise fabrication costs, limit storage stability, and require strict environmental conditions for optimal performance. With the advent of nanotechnology, these constraints have been partly overcome by replacing HRP with peroxidase-like nanozymes. A colorimetric sensor for sarcosine was developed by immobilizing SOx on 2D Fe/Co-MOF nanosheets that exhibit peroxidase-like activity. Bimetallic synergy enhanced catalytic activity, as evidenced by a V_max_ 1.76-fold higher than that of HRP, thereby enabling color development within 20 min and reducing the total analysis time to 30 min. The sensor achieved an LOD of 0.324 μM and a linear range of 1–400 μM, while recoveries in urine samples were in the range of 97.27% to 105.82% and RSDs of 0.4–1.6% [28]. In another work, a distance-based paper analytical device was fabricated using carbon dot peroxidase-like nanozymes. To construct the sensor pad, a TMB solution was applied to the pad surface, while SOx and carbon nanodots were deposited on the sample introduction zone. Urine without prior treatment migrated through the zigzag detection channel by capillary action. When SAR triggered the SOx-nanozyme cascade, a blue colored line developed along the length of the detection channel within 30 min. The distance from the sample zone to the leading edge of the blue-colored signal, measured using a standard ruler, was proportional to SAR concentration of the sample. An LOD of 10.0 nM, extending detectability to the nanomolar range, was calculated from the shortest observable blue line and a linear working range between 12.5 and 35.0 nM was determined. Recoveries in human urine samples were in the range of 98.67 to 101.33% with RSDs < 2.03% [29]. The working range reported is relatively narrow and significantly lower than previously reported clinically relevant concentrations, without clinical data supporting a different range of interest being presented. Recovery experiments using spiked urine for sensor validation do not provide sufficient evidence of the sensor’s applicability to undiluted clinical urine for prostate cancer diagnosis [22,29]. To improve the optical sensors’ analytical performance, alternative labels were selected. Luminol is a low-cost label that was used in a chemiluminescence sensor based on a silver-doped iron metal–organic gel (Ag/Fe-MOG) exhibiting peroxidase-like activity. The activated enzyme cascade produced oxygen reactive species that oxidized luminol. Silver doping of the MOG enhanced its catalytic activity, resulting in a 5.4-fold increase in chemiluminescence intensity and 23 min of analysis time. Detection using a chemiluminescence analyzer gave an LOD of 0.23 mM in a linear range from 1 to 50 mM, whereas visual detection gave an LOD of 1.41 mM. Recoveries in urine were 92.3–115.9% with RSDs below 10%. A charge-coupled device camera was also used to measure chemiluminescence intensity [30]. Although the sensor improved detectability over conventional luminol-based methods, the reported visual LOD is three orders of magnitude higher than the threshold set by Teng et al. [22,30]. Teng et al. 2025 designed a sensor using Fe-MOF MIL-88B(Fe), a peroxidase-like nanozyme, and Au nanorods as labels. The TMB^+^ generated was further oxidized to TMB^2+^ under acidic conditions, which induced etching of the Au nanorods. Due to localized surface plasmon resonance, the changes in the length of Au nanorods alter their color. The sensor achieved an LOD of 0.12 μM with excellent linearity in the range of 1–60 × 10^−6^ M and an analysis time of 15 min. The shortened analysis time is primarily due to the use of Au nanorods, as color development takes 5 min. A smartphone camera was used to capture images for image analysis, while a dual-RGB criterion based on R and B values was proposed for PCa diagnosis. The sensor was finally tested in 14-fold-concentrated urine samples and recovery rates were in the range of 95.52–105.84% with RSDs of 2.4–5.8% [22].

A dual-mode fluorometric/colorimetric sensor was developed utilizing SOx organic–inorganic hybrid nanoflowers, termed Cu_3_(PO_4_)_2_:Ce@SOx. The nanoflowers exhibited fluorescence and peroxidase-like activity. In the presence of SAR and NaOH, the generated H_2_O_2_ oxidized Ce^3+^ to Ce^4+^, resulting in fluorescence quenching at 400 nm and a “turn-on” colorimetric signal by catalyzing the oxidation of TMB into blue TMBox which was monitored at 652 nm. The TMB-based colorimetric mode achieved an LOD of 0.120 µM within a range of 0.18–60 μM, while the fluorometric mode achieved an LOD of 0.226 μM with a dynamic range of 0.5–60 μM and a total analysis time of 45 min. Recoveries were ranging from 93.4% to 107.3% and RSDs of 2.9–9.4%. The sensor’s colorimetric modality was further developed into a cotton swab assay for smartphone detection to facilitate portability and POCT. Linearity was established between grayscale values and the logarithm of SAR concentration (2.44–1250 μM) with an LOD of 1.16 μM. Finally, the method was successfully applied to urine supernatant samples from healthy volunteers [31]. Another dual-mode sensor was developed using fluorescent Fe-doped carbon dots with peroxidase-like activity to oxidize N,N-diethyl-p-phenylenediamine sulfate (DPD). The oxidation product of DPD served as both a chromophore and a fluorescence quencher. Fluorescence quenching was attributed to Förster resonance energy transfer (FRET) and the inner filter effect. The colorimetric mode provided an LOD of 0.17 μM, while the fluorometric mode allowed for an LOD of 0.023 μM over a range of 0.05–120 μM with a total analysis time of 50 min and recoveries of 91.0–109.2% with RSDs below 4.9% [17]. The Fe-doped carbon dot sensor’s LOD in fluorometric mode was approximately 10-fold lower compared to the Cu_3_(PO_4_)_2_:Ce@SOx sensor. In addition, the Fe-doped carbon dot sensor demonstrated a wider detection range in both modalities but required multi-step laboratory procedures. Metal doping enhanced the optical characteristics and energy transfer, thus the overall analytical performance [17,31]. Colorimetric detection has also been complemented by photothermal measurements for signal cross-validation in a dual-mode sensor featuring CuS@CeO_2_ plasmonic nanozymes. The CeO_2_ nanoparticles exhibit peroxidase-like activity which is enhanced by hot-carrier participation, the photothermal effect, and electronic structure modulation. When 2D CuS nanosheets are irradiated by a 1064 nm near-IR laser, they exhibit strong localized surface plasmon resonance that generates hot carriers. A fraction of the hot carrier electrons is transferred from CuS to CeO_2_ and captured by H_2_O_2_. The remaining hot carriers cause thermal dissipation and a local temperature increase, thus improving reaction kinetics. Electronic structure modulation at the interface regulates the Ce^3+^/Ce^4+^ redox ratio and enhances catalytic activity. Colorimetric detection is accomplished using TMB as a chromogenic substrate and measuring absorbance at 652 nm, whereas photothermal detection monitors temperature change using a thermal imager. A smartphone camera was used as an alternative and POCT viable colorimetric sensor. The sensor achieved an LOD of 0.52 μM in colorimetric mode and 0.57 μM in photothermal mode, two linear ranges over a dynamic range of 1–100 μM, and high selectivity and insusceptibility to interference. The sensor was employed for prostate cancer screening of pretreated concentrated clinical urine samples, and the results were compared with histopathological diagnoses. The sensor achieved an AUC of 0.990, a clinical sensitivity of 96.2% and a specificity of 96.4% at the optimal cut-off value of 3.18 μM (that was derived by Youden’s index from ROC analysis). The cut-off value was not validated against an independent cohort to provide an unbiased performance evaluation of the sensor. This also applies to the comparison of the sensor to PSA sensors based on the external training cohort. Moreover, the method validation was limited to a dataset provided by a cohort of 54 people [32]. The previous sensors relied on a SOx-dependent step, which limited further improvements in storage stability and reductions in fabrication costs. Liu et al. 2025 addressed this limitation by developing a SOx-free sensor based on a molecularly imprinted surface Zn/Ce-zeolitic imidazolate framework (Zn/Ce-ZIF) nanozyme. The Zn/Ce-ZIF nanozyme, located at the inorganic core, catalyzed the oxidation of TMB by dissolved oxygen, resulting in a blue color. The molecularly imprinted shell layer facilitated TMB diffusion to the core, while maintaining specificity toward SAR. SAR entered the molecularly imprinted channels that connect the Zn/Ce-ZIF catalytic core to the polymer surface, thereby sterically hindering TMB access to the nanozyme and inhibiting color development. The sensor achieved an LOD of 1.32 μM within the range 2–500 μM in 60 min. Recovery study for two concentrations of SAR spiked in urine showed recoveries of 95.2–106.8% with RSDs 1.9–2.5%. The sensor’s response was also quantified using a smartphone. However, the analysis was more time-consuming than that of enzyme-nanozyme hybrid systems, indicating a trade-off between stability and analytical throughput [33]. A different approach for an enzyme-free sensor was the use of a SOx-mimetic molecule, as implemented by Yang et al. 2025 in a multilayered sandwich nanozyme cascade fluorescence sensor (Cu-MOF/Rf@BDC). Riboflavin (Rf) is analogous to the FAD coenzyme of SOx and exhibits SOx-mimetic activity with high affinity for SAR. Rf fluorescing at 525 nm was in situ embedded within the Cu-MOF. Reaction with SAR induced an electronic state change in Rf, resulting in fluorescence quenching. A peroxidase-like Cu-MOF oxidized intercalated benzene-1,4-dicarboxylic acid (BDC) molecules to produce BDC–OH species fluorescing at 420 nm. The fluorescence intensity ratio at 420 nm and 525 nm was used to detect SAR, with a notably low LOD of 3.31 nM and two-level linear ranges over 0.01–60 μM, over a total analysis time of 30 min. Spiking experiments showed recoveries of 96.71–106.07% with RSD values below 5.00%. The main advantage of the proposed sensor was the confinement-mediated enrichment of H_2_O_2_ inherent in its multilayer design. The sandwich architecture restricted the diffusion of H_2_O_2_ into the bulk solution, thereby increasing the local H_2_O_2_ concentration near the Cu-MOF and enhancing the kinetics of the reaction cascade [34]. Another Rf-based sensor was developed by You et al. 2026 for dual electrochemical/colorimetric detection. Reduced graphene oxide nanoribbons (rGONRs) provided a scaffold for Rf electrodeposition and the encapsulation of Fe(III) meso-tetra(4-carboxyphenyl)porphyrin-Zn MOF (TCPP(Fe)-Zn MOF) with peroxidase-like activity. A key feature of the TCPP ligand was that, owing to its porphyrin structure, it increased the local oxygen concentration and promoted SAR oxidation. o-Phenylenediamine was used both as an electroactive species and a chromogenic substrate. Electrochemical detection was performed through CV and EIS with an LOD of 0.04 nM over a detection range of 0.001–500.0 μM. The colorimetric mode used smartphone-based RGB analysis with an LOD of 2.0 μM over a detection range of 10–1000.0 μM and recoveries in urine samples of 97.1–110.7% and RSD about 4.2% for the colorimetric and 5.3% for the electrochemical sensor [35]. A cellulose nanofiber/polyacrylic acid (CNF/PAA) hydrogel-based colorimetric sensor incorporated into a 3D-printed strip was also fabricated for SAR detection within 30 min. The detection was again based on SOx and TMB and showed an LOD of 10 μM with a linear range of 0–100 μM. The sensor was also tested in artificial urine samples devoid of creatinine [36].

Summarizing SAR detection by smartphone-derived colorimetric sensors, most reports include the following stages: (i) sample pre-treatment, (ii) enzymatic SAR oxidation, (iii) color development and (iv) smartphone signal reading. Sample pre-treatment usually includes centrifugation, filtration and dilution to minimize interferences from urine. EDTA can also be used to block metal ion interference. Then SAR is oxidized by SOx or SOx-mimicking nanozymes, a reaction that requires an alkaline or near-neutral environment for 25 to 30 min to convert SAR into glycine, formaldehyde, and H_2_O_2_. Then, a peroxidase-catalyzed reaction using the produced H_2_O_2_ is performed converting chromogens (such as 3,3′,5,5′-tetramethylbenzidine [TMB] or o-phenylenediamine [OPD]) into colored oxidized products. This reaction requires acidic conditions and 5–10 min for color development. Finally, image capture by a smartphone relies on external illumination boxes equipped with LED lights, fixed focal distances and mobile image analysis software that extracts RGB or grayscale values and correlates optical density to concentration.

Valine and leucine have also been proposed as biomarkers for PCa when detected at elevated levels as unbound amino acids [37,38]. A study on PCa diagnosis based on amino acid profiling of urine using HPLC demonstrated that leucine provided the highest discriminatory performance with an AUC of 0.79 [39]. Fluorescent L-cysteine-capped copper nanoclusters (L-cys@CuNCs) were used for detecting valine. Fe^3+^ ions interacted with the cysteine-capped surface via thiol and carboxyl groups, resulting in fluorescence quenching. Fluorescence intensity increased in response to valine, possibly due to competing for coordination with Fe^3+^. Valine was detected at an LOD of 3.0 μM over a range of 73–366 μM. Recovery was achieved in the range of 94.85–101.16% [38]. A similar sensor was developed for detecting leucine. Fluorescent Cu–AgNCs were employed in place of L-cys@CuNCs, achieving an LOD of 0.58 μM and a linear range of 110–657 μM and recoveries of 84.9–100.6% [37]. The mechanistic uncertainty in fluorescence emission warrants further investigation and suggests the need for more extensive specificity tests to assess the sensor’s selectivity. Cysteine is a biomarker associated with cervical cancer and present in the urine of patients at concentrations of 190–260 μM. Yang et al. 2026 developed a dual colorimetric/fluorescence xanthyllium-based sensor for cysteine detection in urine. Cysteine cleaves the nitrobenzoxadiazole ether moiety of the xanthyllium-based probe SH-OH-NBD through nucleophilic aromatic substitution. The product of a subsequent rearrangement reaction generates a new absorption peak at 490 nm and a fluorescence emission peak at 567 nm. Upon addition of cysteine to the sensing solution, color gradually changed from purple to pale yellow and the color change after 1 h was captured using a smartphone camera. Smartphone-assisted quantification with a color analysis app, and an RGB-based criterion achieved an LOD of 31.8 μM and a linear range of 0–300 μM. Fluorescence mode improved sensitivity, demonstrating an LOD of 3.1 μM over the same concentration range. Colorimetric mode achieved recoveries of 90.7–106.3% and RSDs of 7.92–10.11%, whereas fluorometric mode was more accurate and repeatable achieving recoveries of 97.3–99.9% and RSDs of 0.49–1.16%. The probe also exhibited good solubility in aqueous solutions and selectivity [40]. Another metabolite associated with PCa is citrate. Studies investigating urinary metabolomics have shown altered urinary citrate concentrations in patients with bladder cancer. Several studies have reported lower citrate concentrations compared with healthy controls, supporting its potential as a non-invasive metabolic biomarker for bladder cancer [41]. This systematic review specifically identifies urinary citric acid among the metabolites associated with bladder cancer and reports its consistent downregulation. Prostate fluid with citrate concentration decreased to the range of 2–20 mM is indicative of malignancy. Zhu et al. 2025 obtained N-doped CDs via biogenic synthesis using a biowaste substrate, *Syzygium cumini* seeds, aiming to develop low-cost fluorescent probes. Citrate interacted with the N-doped CDs, likely via hydrogen bonding and electrostatic interactions, thereby quenching fluorescence. The sensor achieved an LOD of 3.5 nM, retained selectivity in the presence of potential interferents, and required a total analysis time of 5 min. Accuracy and reproducibility were excellent with recoveries achieved in the range of 99.3–100.07% and RSDs of 0.91–1.02%. Importantly, the sensor exhibited low cytotoxicity, enabling bioimaging [42].

In addition to enzymes and nanozymes as recognition elements for metabolite detection in urine, molecularly imprinted polymers (MIPs) have also been explored. A fluorescence sensor was developed based on N,S co-doped carbon dots on a silica surface encapsulated within a molecularly imprinted polymer (N,S-CDs@SiO_2_@MIPs) for 4-Hydroxybenzoic acid (4-HBA). The rapid development of metabolomics has identified 4-HBA in urine as a crucial biomarker for the early detection and screening of gastric cancer [43,44]. 4-HBA was bound to the MIP, resulting in fluorescence quenching via dynamic quenching and electron transfer. An LOD of 1.27 μM was achieved within the dynamic range of 0–100 μM with a total analysis time of 18 min, and an imprinting factor (IF) of 3.32. Recovery rate in urine was 97.87–113.63% with RSDs < 2.89% and a total analysis time of 18 min [43]. Another fluorescence sensor for 4-HBA was developed by Jie et al. 2026 based on ZIF-8 and CD nanocomposite, encapsulated within a silica shell and embedded in a MIP (ZIF/CDs@MIPs). Incorporation of ZIF-8 reached an LOD of 0.68 μM, enhanced molecular recognition, as evidenced by an IF of 4.35, and improved the overall stability of the sensor compared to the previous study. In addition, the optimal response time was reduced to 13 min. However, the sensor exhibited a narrower detection range (0–100 μM). Spiking experiments showed recovery rates of 97.71–106.16% and low RSDs of 0.80–2.95% [10]. Moreover, a fluorescence sensor for 4-HBA detection, developed by Jie et al. 2025, was based on magnetic hollow carbon dots incorporating Fe_3_O_4_ nanoparticles, which were embedded in an MIP (Fe_3_O_4_/CDs@MIPs). Incorporation of Fe_3_O_4_ nanoparticles improved the sensor’s analytical performance, lowering the LOD to 0.37 μM and reducing the total analysis time to 11.5 min within the concentration range of 0–100 μM. The sensor exhibited an imprinting factor (IF) of 40.78, an unusually high value, explained by a low non-imprinted polymer baseline and recoveries in the range of 95.00% to 106.67% and RSDs between 0.76% and 2.80% [44]. Vanillylmandelic Acid (VMA) is a well-characterized urinary catecholamine metabolite used in the adjunctive diagnosis of neuroblastoma. More recent studies continue to evaluate VMA alongside other urinary catecholamine metabolites in diagnostic and pretreatment risk-assessment models for neuroblastoma [45]. Meng et al. 2024 developed a luminescence sensor based on planar 14-metal Tb(III) nanoclusters for detecting VMA. VMA enhanced the fluorescence of the 14-metal Tb(III) nanocluster via the antenna effect, resulting in a dark green color. The sensor achieved LODs of 0.32 nM in acetonitrile and 0.34 nM in urine with a rapid response time of less than 10 s. Recoveries and RSDs were 98.73–100.03% and 3.34–8.55%, respectively. Test strip implementation of the sensor enabled visual detection under UV light. Importantly, the sample application to the test strip without pre-treatment expedited the analytical process [46]. A different metal nanocluster was used for the detection of biopterin, a biomarker associated with various cancer types. Biopterin, specifically 6-biopterin, has been investigated as a potential non-invasive metabolic biomarker for cancer, with elevated urinary concentrations reported in cancer patients compared with healthy individuals [47]. The synthesized 19-metal Cd(II)−Eu(III) nanocluster responds to biopterin via the antenna effect, leading to an enhancement of Eu(III) fluorescence and a red color shift. Fluorescence intensity measurements using simulated urine samples achieved an LOD of 0.11 nM in CH_3_CN, 0.21 nM in fetal calf serum and 0.22 nM in urine over a linear range of 0–100 μM, recovery rates of 95.6–108.2% and RSDs of 1.64–6.08% in CH_3_CN, recovery rates of 96.17–109.79% and RSDs of 0.26–1.23% in fetal calf serum, and recovery rates of 94.05–113.73% and RSDs of 0.34–1.43% in artificial urine. Test strips were fabricated by drying a solution of the metal nanocluster onto filter paper. The test strips were used for quantitative detection with smartphone achieving recovery rates of 97.47–109.33% [48].

#### 2.2.2. Proteins

Proteins are promising biomarkers for early cancer detection. However, reliable sensing systems are still in demand [21]. Protein biomarkers consist of circulating tumor-associated proteins that are released either directly by cancer cells or indirectly as a consequence of tumor-induced systemic responses. This category includes a broad range of molecules, such as tumor antigens, enzymes, cytokines, chemokines, and other soluble mediators, which can be detected in various biological fluids. Circulating protein biomarkers represent some of the most well-established diagnostic tools in oncology. Representative examples include prostate-specific antigen (PSA), cancer antigen 15-3 (CA 15-3), carbohydrate antigen 19-9 (CA 19-9), carcinoembryonic antigen (CEA), and alpha-fetoprotein (AFP), which are used in the detection of several cancer types. However, despite their established clinical applications, individual protein biomarkers frequently exhibit limited sensitivity and specificity. More recently, panels of multiple protein biomarkers have emerged as a promising approach to improve screening and diagnosis. By integrating complementary biomarker information, such panels may reduce both false-negative and false-positive results while providing greater predictive value [5]. An immunoassay using a carboxy-porous organic polymer as a fluorescent probe was developed for detecting prostate-specific antigen (PSA) in urine. The porous organic polymer was synthesized from 2,4-dihydroxybenzenetricarbaldehyde (DHBTC) and 3,5-diaminobenzoic acid (DAB). Subsequently, a monoclonal antibody specific to PSA was conjugated to the DHBTC-DAB polymer via EDC/NHS chemistry. Antibody-PSA interaction altered the electronic environment of DHBTC-DAB, resulting in static fluorescence quenching by the formation of a non-emissive ground-state complex. The sensor achieved an LOD of 14.5 pg/mL over a detection range of 0–36 ng/mL and a total analysis time of 4 min. Spiking of the protein in urine in three concentrations showed recoveries of 95.7–104.0% and RSDs of 0.16–1.70% [21]. A ‘turn-on’ fluorescence sensor utilizing fluorescent copper nanoclusters (CuNCs) and graphene oxide (GO) was designed for detecting glial fibrillary acidic protein (GFAP), a biomarker of glioblastoma and other astrocytic malignancies. L-cysteine-capped CuNCs were conjugated with antibodies specific to GFAP via EDC/NHS chemistry. Because of FRET, GO quenched the fluorescence of the CuNCs. The binding of GFAP to the conjugate induced conformational changes that restored fluorescence due to steric hindrance between the CuNCs and the GO. The sensor achieved an LOD of 32.0 pg/mL over a detection range of 0.0–46.3 ng/mL. The sensor was suitable for various biological fluids, namely saliva, serum, and urine, with recoveries of 84.8–100.4% [49].

In addition, lateral flow assays (LFAs) offer rapid analysis times, low per-assay costs, and an operationally simple workflow. LFAs have found particular use in point-of-care applications for disease diagnosis. An LFA was developed for detecting urinary cancer biomarkers, such as chemokine ligand 9 (CXCL9) and chemokine ligand 10 (CXCL10). The working principle was initially implemented in a fluorescence immunoassay and was subsequently adapted into a lateral flow device. Target biomarkers were pre-concentrated using magnetic beads coated with capture antibodies. A different antibody conjugated to a DNA barcode complex containing Cas12a recognition sites bound to a different epitope of the analyte, thereby forming a sandwich complex. Signal amplification was achieved through Cas12a-mediated trans-cleavage of oligonucleotide reporters. In the fluorescence assay, the FRET-based reporters were non-fluorescent until they were cleaved by activated Cas12a. The fluorescence immunoassay achieved LODs of 0.6 pg/mL for CXCL9 and 2.4 pg/mL for CXCL10, with a total analysis time of 90 min. A commercial LFA strip was repurposed as a detection platform for the CRISPR-amplified sensor response. Reporter molecules were labeled with a fluorophore at the 5′ end and a biotin group at the 3′ end. In this strip design, the control zone preceded the test zone in the flow direction. Streptavidin was immobilized at the control zone, and secondary antibodies were immobilized at the test zone. In the absence of the target analyte, reporter molecules remained intact and were captured in the control zone. Gold nanoparticles conjugated to anti-fluorophore antibodies bound to the intact reporter molecules retained at the control zone, resulting in the appearance of a visible red line. In the presence of the target analyte, the signal intensity in the control zone was suppressed, and gold nanoparticles were captured in the test zone by immobilized secondary antibodies. The LFA device achieved LODs of 18 pg/mL for CXCL9 and 39 pg/mL for CXCL10, with a total analysis time of 93 min [50].

Another LFA was designed by Amparore et al. 2025 as part of a dual-visual sensor to detect PSA and zinc in urine. Its analytical performance was evaluated using urine samples collected from men following a standardized prostatic massage. Threshold values were used to distinguish between distinct diagnostic risk categories (2800 ng/mL for PSA and 1400 ng/mL for Zn). PSA was detected using a competitive LFA, whereas urinary zinc was detected using a colorimetric assay based on a dithizone-saturated membrane as cancer disrupts Zn accumulation in prostate tissue. The model’s analytical performance was validated in a study of 242 patients, with an area-under-curve (AUC) value of 0.769 on the Receiver Operating Characteristic (ROC) curve. The dual PSA-zinc sensor was proposed for early cancer detection, as it can detect PCa in cases deemed inconclusive by conventional methods [51]. Finally, Ni et al. 2025 developed a conductivity-regulated bipolar electrochemiluminescence platform for detecting hyaluronidase, a biomarker associated with bladder cancer. The platform consists of a sensing cell and a reporting cell. In the sensing cell, hyaluronidase degraded hyaluronic acid, releasing negatively charged fragments that exhibited enhanced electromigration rates, thereby increasing the conductivity of the sensing solution. The increase in conductivity was translated to a voltage drop across the bipolar electrode, promoting electron transfer from tris(2,2′-bipyridyl)ruthenium(II) ([Ru(bpy)_3_]^2+^) toward the anode, resulting in electrochemiluminescence emission. On this platform, the addition of an exogenous electroactive indicator is unnecessary, unlike in most electrochemical methods, thereby simplifying the system and avoiding interference with hyaluronidase activity. The platform achieved an LOD of 0.13 U/mL over a range of 1–60 U/mL with a total analysis time of 90 min [52].

#### 2.2.3. RNA Nucleosides

The hypoxia and necrosis induced by uncontrolled cell proliferation of tumors results in adenine nucleotide degradation and release of adenosine, providing a favorable environment for tumor growth. The conversion of adenine nucleotides to adenosine is catalyzed by multiple enzymes either in the cytosol or extracellularly [53]. Adenosine levels were increased in urine of lung and colorectal cancer patients; therefore, it has been successfully used by sensing platforms as an important nucleoside for cancer diagnosis [54,55]. Sun et al. 2025 developed an aptasensor-based chemiluminescence platform using luminol as a label for detecting adenosine. Fe_3_O_4_@nSiO_2_ particles were synthesized and conjugated with DNAH1, a hairpin aptamer for adenosine. Au@ZIF67 nanoparticles were functionalized with ssDNA and hybridized via complementary base pairing with DNAH1. The binding of adenosine to DNAH1 released the nanozyme Au@ZIF67, generating reactive oxygen species that oxidized luminol. Chemiluminescence intensity was amplified through a catalytic hairpin assembly. DNAH2, a hairpin DNA strand complementary to DNAH1, displaced adenosine from DNAH1 to form a DNAH1-DNAH2 duplex. The displaced adenosine initiated additional cycles of Au@ZIF67 release through target recycling. The sensor achieved an LOD of 1.5 pM over a detection range of 10 pM–100 nM, recoveries of 97.6–101.7% and a total analysis time of 90 min [56]. Furthermore, dysregulated RNA turnover in cancerous cells promotes increased intracellular levels of modified nucleosides. Modified nucleosides cannot be incorporated in tRNA biosynthesis via the salvage pathway, and they are excreted in urine [57,58]. Chemometrics and artificial neural network analysis have been used for cancer diagnosis from the profile of modified nucleosides [59,60]. N^1^-methyladenosine (m^1^A) is a urinary biomarker associated with cancer development and tumor progression. Bağda et al. 2025 developed a strand-displacement-based fluorescence sensor for m^1^A detection. An aptamer specific to m^1^A was labeled with carboxyfluorescein and hybridized with a complementary DNA strand conjugated to a fluorescence quencher. In the presence of m^1^A, the quencher-conjugated strand was displaced to produce fluorescence. The sensor achieved an LOD of 1.9 μM over a detection range of 0–25 μM, with a total analysis time of 30 min. Recovery values were in the range of 86–116%. The sensor demonstrated operational simplicity and specificity by direct application of urine samples to the sensing solution [61]. A tetralactam macrocycle-based fluorescent indicator displacement assay was also developed for detecting 16 RNA nucleosides. The target RNA nucleosides included unmodified, methylated, and other chemically modified derivatives. A water-soluble naphthalene-based endo-functionalized tetralactam macrocycle, termed H1, served as the recognition element. H1 exhibited distinct binding affinities for each target analyte, reflecting differences in hydrophobicity and hydrogen-bonding capacity. Two fluorescent dyes, methylene blue and 2-(4-dimethylaminostyryl)-1-methyl-pyridinium iodide (2-ASP), bound within the H1 cavity with different binding affinities. The fluorescence of methylene blue was quenched upon binding to H1, whereas the fluorescence of 2-ASP was enhanced. Target analytes competitively displaced the fluorescent dyes from the H1 cavity. Following optimization studies, a three-element sensor array was fabricated, comprising three distinct host-dye pairings at varying ratios, thereby producing a unique signal fingerprint for each analyte. For adenosine, the sensor achieved an LOD of 1.1 μM over a detection range of 0–60 μM. The sensor demonstrated 100% accuracy when distinguishing between target nucleosides. For multiplexed detection, the sensor quantified simultaneously three target analytes in a ternary mixture using support vector regression. The sensor was validated in simulated urine samples representative of breast, colorectal, and urogenital cancer patients [62].

#### 2.2.4. Nucleic Acids

Nucleic acids are usually the preferrable analytes due to their stability in various conditions and their detection even at very low concentration using amplification techniques, especially when compared to proteins. Circulating tumor DNA (ctDNA) fragments contain different mutations than cell-free DNA, being excellent diagnostic and prognostic biomarkers of tumor and therapy monitoring due to their excellent correlation with the molecular pathology of the tumor. MicroRNAs are small molecules responsible for many cell functions as gene expression, thus they have emerged as excellent liquid biopsy biomarkers. Their analysis is usually performed through PCR- and RT-PCR-based techniques and DNA/RNA sequencing. These techniques, however, are expensive, while sequencing results require a long time to be communicated back to the clinicians [63].

Xiang et al. 2024 developed an in vivo sensing technique using a probe functionalized with DNA/γ-modified peptide nucleic acid (PNA) for fluorometric detection of ctDNA, namely the KRAS proto-oncogene carrying the G12D mutation (KRAS(G12D)). γ-Modified PNA complementary to the KRAS(G12D) ctDNA sequence was labeled with the fluorophore cyanine 5 (Cy5) and hybridized with a complementary DNA strand, termed the DNA linker. The DNA linker was modified with Black Hole Quencher 3 (BHQ3) at the 3′ end. The fluorescence of Cy5 was quenched due to FRET. The hybridized DNA linker was conjugated to an 8-arm poly(ethylene glycol) (PEG) nanoparticle via click chemistry. Additionally, the 8-arm PEG core was conjugated with a 1,2-distearoyl-sn-glycero-3-phosphoethanolamine-N-[amino(polyethylene-glycol)-2000]-DBCO albumin hitchhiking module that extended the nanoreporter’s half-life during blood circulation to 1.3 h. The resulting nanoreporter was designated as Lpeg-LPp. Lpeg-LPp was intravenously injected into pancreatic ductal adenocarcinoma-bearing mice. Through the circulatory system, Lpeg-LPp targeted the KRAS(G12D) ctDNA. The target ctDNA displaced the γPNA from the linker DNA strand-nanoparticle complex via toehold-mediated strand displacement, forming a γPNA/ctDNA duplex. Spatial separation of Cy5 from BHQ3 produced fluorescence. The liver cleared the BHQ3-labeled nanoparticles, while γPNA/ctDNA duplexes were excreted in urine due to renal size filtration. The fluorescence signal was amplified via a hybridization chain reaction (HCR), achieving an LOD of 5.4 pM over a range of 30–1000 pM, with a total analysis time of 5.75 h. The method was applied to different types of tumor cell DNA extracts (including MCF-7 breast cancer cells, A549 lung cancer cells and PANC-1 cells) and to mouse tumor models. Testing revealed that the sensing platform was specific to the G12D mutation, the nanoreporter exhibited negligible cytotoxicity, and liver and kidney functions were not affected. The main limitations of the method are the lack of clinical trials in human patients, the mildly invasive administration of the nanoprobe, and an LOD that may not be sufficient for very early cancer detection [63]. A sandwich-type LFA was developed for detecting mRNA cancer biomarkers, namely PSA and Prostate Cancer Antigen 3 (PCA3). The mRNAs were amplified isothermally at body temperature using a polydimethylsiloxane reaction chamber via reverse transcription-recombinase-aided amplification. Forward primers were labeled with biotin, while reverse primers were labeled with fluorescein isothiocyanate (FITC) to produce dual-labeled amplicons. Streptavidin was immobilized on the test line, and secondary anti-rabbit antibodies were immobilized on the control line. Anti-FITC-functionalized gold nanoparticles (AuNPs) served as visual reporters and analysis was completed within 20 min. LFA accurately distinguished negative controls from PCa patient urine in 20 clinical samples [64].

As for microRNAs detection, a colorimetric deoxyribozyme-based assay was developed for the breast cancer biomarkers miRNA-21 and miRNA-155. Two probes, termed P1 and P2, were designed, each containing an intermediate sequence complementary to miRNA-155 or miRNA-21, respectively, and two guanine-rich regions. In the absence of P1, P2 forms a stem-loop structure and vice versa. When the probes were mixed and denatured, single-stranded molecules were formed. In the absence of either miRNA target, the probes hybridized into a G-quadruplex (G4) configuration. G4 exhibited peroxidase-like deoxyribozyme activity when hemin, NH_4_^+^, and K^+^ cations were present. TMB was used as a chromogen, resulting in blue coloration of negative samples. When either target miRNA was present, it hybridized with its complementary probe, preventing G4 formation. The sensor achieved an LOD of 0.43 nM in buffer and 0.62 nM in urine over a range of 0–10 nM, with a total analysis time of 65 min [65]. Next, a novel approach is examined for the separate detection of a nucleic acid and a protein biomarker using the same analytical platform. Wang et al. 2025 developed a DNA nanoswitch-regulated CRISPR/Cas12a-gold nanostar reporter platform for detecting miRNA-375 and PSA, two PCa biomarkers. Gold nanostars@SiO_2_ were functionalized with single-stranded DNA (ssDNA). ssDNA carried the fluorescent label Cy5.5 at the end adjacent to the Au nanostars and the quencher BHQ3 at the opposite end. The spatial confinement effect of ssDNA on the surface of Au nanostars accelerated reaction kinetics over previous methods. The intact reporter nanoparticle did not fluoresce. Two hairpin DNA molecules, termed DNA nanoswitches, were designed to be complementary to miRNA-375 and an aptamer sequence for PSA, respectively. The platform enabled the detection of either target analyte using the appropriate nanoswitch. When the DNA nanoswitch recognized the target analyte, it triggered a catalytic hairpin assembly, releasing a double-stranded DNA activator. The activated Cas12a cleaved the ssDNA strands conjugated to the Au nanostars, producing plasmon-enhanced fluorescence. The sensor achieved an LOD of 17.3 fM for miRNA over the range of 0.05–20 pM and an LOD of 27.7 pg/mL for PSA over the range of 0.1–50 ng/mL. The total analysis time was 135 min. The platform was validated with clinical urine samples from a cohort of *n* = 20 and exhibited 99% diagnostic accuracy through principal component analysis using correlation analysis with RT-PCR for miRNA-375 and ELISA for PSA [66]. An analytical platform/chip was also developed for the diagnosis of urethral carcinoma, using a droplet-based digital exponential amplification reaction (ddEXPAR) for detecting the EV-derived miRNAs let-7a, miR-223, and miR-143. The amplification process was conducted within thousands of femtoliter-sized hydrogel-based microdroplets and cross-linked with tetra-armed poly(ethylene glycol), acrylate (Tetra-PEGA) and poly(ethylene glycol) dithiol (HS-PEG-SH) for microRNA quantification. Urine was filtered, and EVs were isolated using a dedicated kit with anti-CD63 antibody-functionalized magnetic beads. EV lysis was followed by multiplexed ddEXPAR of the released miRNAs within a hydrogel matrix, thereby confining the amplification reaction to a femtoliter-sized reactor volume. Three ssDNA dyes were used to distinguish between analytes: SYBR Green for let-7a EXPAR products, SYTOX Blue for miR-223, and SYTOX AADvanced for miR-143. Digital counting of fluorescent microdroplets enabled quantitative detection. The platform achieved a dynamic range of 50 fM–30 nM in ternary miRNA mixtures. The AUC-ROC values for discriminating against cancer patients from healthy individuals were 0.85, 0.89, and 0.92 for the three miRNAs, respectively. The method lowered the detection limit to the femtomolar scale (3 fM) and the sample volume, limited non-specific amplification, as the hydrogel framework offered excellent permeability for the free diffusion of nucleic acids and removal of unreacted primers to avoid nonspecific cross-reactions, achieved a cost of 3.5$ per assay, enhanced simplicity and reduced analysis time to 50 min, presenting numerous improvements over commercial ddPCR techniques [67]. Finally, Park et al. 2024 designed an all-in-one fusogenic nanoreactor (FNR) for FRET-based detection of EV-derived miR-200c, miR-222, and miR-375 to aid diagnosis of breast cancer. Typical analysis of EV components includes qPCR or RT-PCR that are time-consuming and of high cost. Alternative biosensors and microfluidic devices have emerged to enable rapid, low-cost and multiplex analysis of EV-derived biomarkers The all-in-one FNR accomplishes single-step detection, circumventing the standard RNA extraction, reverse transcription, and qPCR workflow. The FNR encapsulates a three-stranded DNA-fueled machine comprising a template strand, a donor probe, and an acceptor probe. The template strand is complementary to the target miRNA and labeled with Cy3. The donor probe is labeled with Cy3, and the acceptor strand is labeled with Cy5. Following activation with trypsin and a pH of 5.2, the FNR induces membrane fusion with extracellular vesicles containing the target miRNAs via haemagglutinin-sialic acid recognition. FRET reduction is accomplished via toehold-mediated strand exchange, resulting in the release of the acceptor strand and changing the FRET ratio. Release of the acceptor probe exposed a second toehold complementary to the fuel strand, triggering a second toehold-mediated strand exchange that released the target miRNA, thereby amplifying the signal. The FNR was tested using orthotopic breast cancer mouse models. Collected urine was centrifuged and filtered, and extracellular vesicles were isolated using a commercial kit. The LOD was 1.42 × 10^4^, 2.95 × 10^3^, and 4.23 × 10^4^ EVs/mL for MCF-7 EVs (target: miR-200c), SKBR-3 EVs (target: miR-200c), and MDA-MB-231 EVs (target: miR-222), respectively. Using multivariate analysis, the sensor achieved a 75% discrimination accuracy between healthy and cancerous mouse models with an AUC-ROC of 0.81. When interpreting the results, it should be considered that they were derived from a small number of animals (*n* = 10), and train–test set splitting at the animal level, whereas external validation was not conducted. The lack of testing in human urine and the relatively low discrimination accuracy highlight limitations that could guide further development of the FNR. The FNR enabled simultaneous detection of all three analytes by performing parallel analyses in three different wells. The confined reaction volume provided by EVs fused with the FNR reduced signal acquisition time to 30 min, yielding a total analysis time of 60 min for urine samples [68].

#### 2.2.5. Extracellular Vesicles and Exosomes

Extracellular vesicles (EVs), including exosomes, are secreted at high concentrations into body fluids and play a crucial role in between-cells communication, therefore they are considered as a significant source of circulating cancer biomarkers in liquid biopsy applications. EVs carry a specific panel of miRNAs, RNAs, other small RNA molecules and ctDNAs that comprise the molecular identity of the parental tumor, providing valuable information for the tumor [68]. In recent years, research has also turned to exosomal products, such as nucleic acids, proteins, lipids, and metabolites in the field of liquid biopsy. Exosomal proteins, in particular, have a role in the formation of the cancer microenvironment, tumor progression, and metastasis [4]. Several studies have shown that specific metabolites, such as lactate, myoinositol, and others, have been considered as possible cancer urine biomarkers that exhibit changes in the concentration levels in case of cancer, especially in renal cell carcinoma, a urinary cancer [69,70,71].

A low-cost, equipment-free, and easy-to-use colorimetric sensor based on anti-HER2-functionalized magnetic polydiacetylene nanoparticles (anti-HER2@MPDA) was developed for detecting HER2-overexpressing EVs, a breast cancer biomarker. HER2-overexpressing EVs bound to the anti-HER2@MPDA and were separated using a magnetic field. The affinity between HER2 and anti-HER2 induced a conformational change in the nanoparticles’ PDA backbone, resulting in a color shift from blue to red. The sensor was evaluated using mouse urine samples from a HER2-overexpressing breast cancer mouse model, achieving an LOD of 8.5 × 10^8^ particles/mL and a total analysis time of 2 h [72]. A high-throughput exosome detection system was developed by integrating encoded microcarriers-photonic crystal microparticles that have enhanced affinity for exosomes and distinctly colored barcode hydrogels- with herringbone microfluidic technology for multiple exosomal biomarker detection based on specific antibodies conjugated to the hydrogels. FITC was used as reporter for fluorescent detection and quantification using a fiber-optic spectrometer. The platform exhibited a detection range of 10^3^ to 10^8^ particles/mL with an LOD of 10^3^ particles/mL. By simultaneously analyzing 6 biomarkers of urine-derived EVs, the ROC analysis showed an AUC of 0.8 that is in agreement with ELISA results [15]. A polydimethylsiloxane (PDMS) optofluidic biosensor, a fully automated integrated system, was developed for the on-chip sample processing and the optical detection and counting of single EVs. SYBR Green was used as reporter. The EV concentration was measured within 30 s after isolation of EVs from urine. The system was also integrated with a Raspberry Pi and IoT for remote control [73].

### 2.3. Surface-Enhanced Raman Spectroscopy (SERS)

Surface-enhanced Raman spectroscopy (SERS) has emerged as a highly sensitive analytical technique that also provides quantitative data. SERS is based on the localized surface plasmon resonance effect of metallic plasmonic nanoparticles, e.g., silver and gold nanoparticles, which enhance the optical fingerprints achieved using SERS and enable the quantitative detection of single molecules even at very low concentrations, as well as improving the reproducibility of the technique. SERS has been introduced as an optical liquid biopsy technique by detecting biomarkers in urine. In addition, low-cost, label-free, and noninvasive surface-enhanced Raman spectra have become ideal analytical tool for enabling point-of-care diagnostic techniques [74,75].

#### 2.3.1. microRNAs

**Figure 1:**
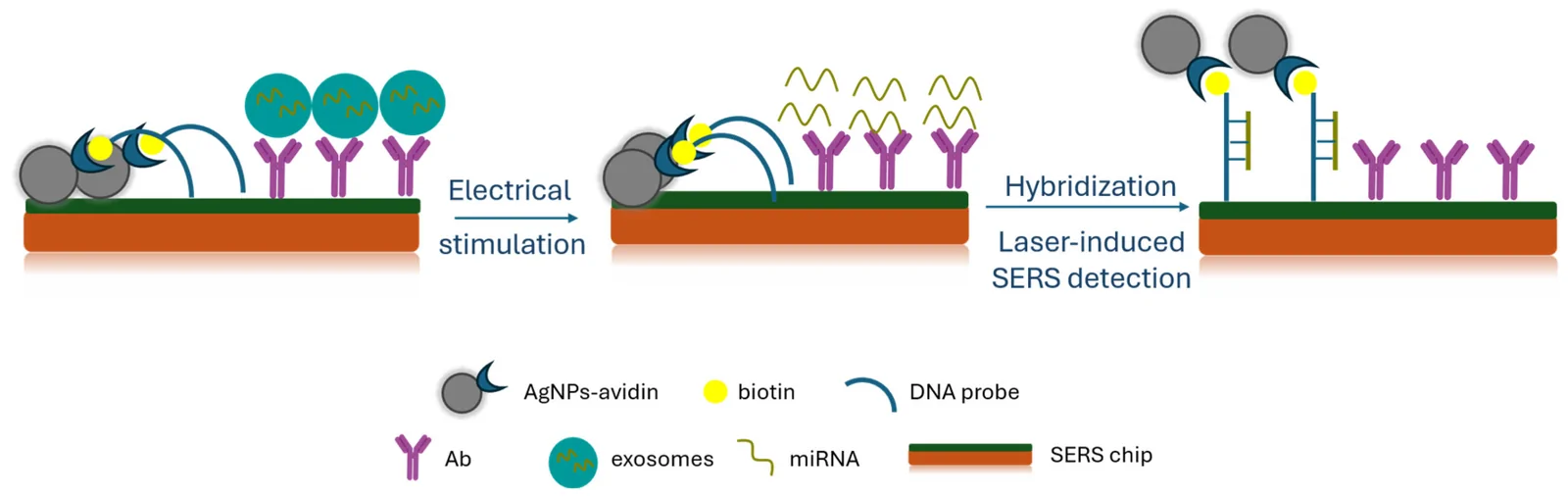
An electrically activated SERS nanoplatform for the detection of exosomal miRNA molecules. Exosomes are captured through specific antibodies immobilized onto an ITO electrode’s surface. The exosomes are then lysed through the application of a slight electric field. Released miRNAs are subsequently captured to immobilized detection DNA probes that are attached to avidin-AgNPs. Upon hybridization, AgNPs are released from the electrode’s surface enabling SERS measurement. AgNPs: silver nanoparticles, Ab: antibody.

A novel approach used an electrically activated SERS nanoplatform for the rapid detection and identification of exosomal miRNAs. The principle involved a novel miRNA release method from exosomes that were captured on an ITO electrode’s silver surface coated with exosome-specific antibodies using a slightly applied electric field and subsequent SERS-based detection. MicroRNA molecules were captured to immobilized DNA probes conjugated to silver nanoparticles enabling SERS signals (Figure 1). The developed sensor chip provided excellent specificity with an LOD of 0.5 nM and was successfully applied to urine and blood serum samples. The proposed sensor demonstrated the ability to detect higher miRNA content in cancer-derived exosomes isolated from complex body fluids compared to normal cells [20].

#### 2.3.2. Other Biomarkers

A novel method reports a 3D DNA hydrogel containing bimetallic Au@Ag nanoparticles and nitrogen-doped quantum dots on graphene oxide (GO) for the rapid dual-mode detection and quantification of epithelial cell adhesion molecule (EpCAM), a broad-spectrum biomarker. Urinary EpCAM has been investigated as a non-invasive biomarker for urothelial bladder cancer. Elevated urinary EpCAM levels have been associated with increasing tumor stage [76]. SERS in combination with fluorescence signals is one of the favorite dual-mode systems due to rapid analysis and high detectability. The principle relies on an aptamer DNA in the hydrogel that interacts with EpCAM, which causes disassembly of the DNA hydrogel and simultaneously switches the SERS signal from “on-to-off” and the fluorescence signal from “off-to-on”. The method had a linear range of 0.5 to 60.0 pg/mL with LODs of 0.17 pg/mL for SERS and 0.35 pg/mL for fluorescence. The method was applied to cell lysates (Hela, A549, MCF-7), human serum, and clinical urine samples from healthy individuals and renal injury patients. When applied to urine samples (*n* = 3) the sensor exhibited recoveries ranged from 85.8–98.3% and (relative standard deviations) RSDs ranging from 2.4–5.7% [16]. Another method focuses on the early-stage diagnosis of bladder cancer through label-free SERS analysis of nano-biomarkers in urine. The principle involves depositing a single 5-μL urine drop onto a Au-coated ZnO nanoporous SERS chip for 30 min to allow for in-situ filtration of biomarkers into nanoscale cavities, followed by Raman spectral acquisition using a 785-nm laser. The method was applied to rat urine samples obtained over multiple time points to monitor cancer progression. The combination of SERS and machine learning (primary component analysis–partial least squares discriminant analysis) achieved an accuracy ≥99.6%. The dataset (*n* = 240) was split 80:20 into a training set and a test set, and five-fold cross-validation was performed. [75]. Finally, a novel sensor for bladder cancer was proposed, using hyaluronic acid-coated silver nanoparticles (HA-AgNPs) as a substrate to detect hyaluronidase. Upon oxidation of hyaluronic acid of the above conjugates by hyaluronidase, oligomers are released that cause aggregation of the nanoparticles and therefore lower SERS signal. SERS signal was based on rhodamine. The sensor had a linear range of 3 × 10^−4^ U/mL to 3 × 10^1^ U/mL, while the analysis of 42 normal and 40 bladder cancer urine samples combining principal component analysis (PCA) with linear discriminant analysis (LDA) and using the leave-one-out cross-validation method showed an accuracy of 95.1%, a sensitivity of 95.2% and a specificity of 95% with RSDs < 5.47% [77].

### 2.4. Surface Plasmon Resonance (SPR) Sensors

**Figure 2:**
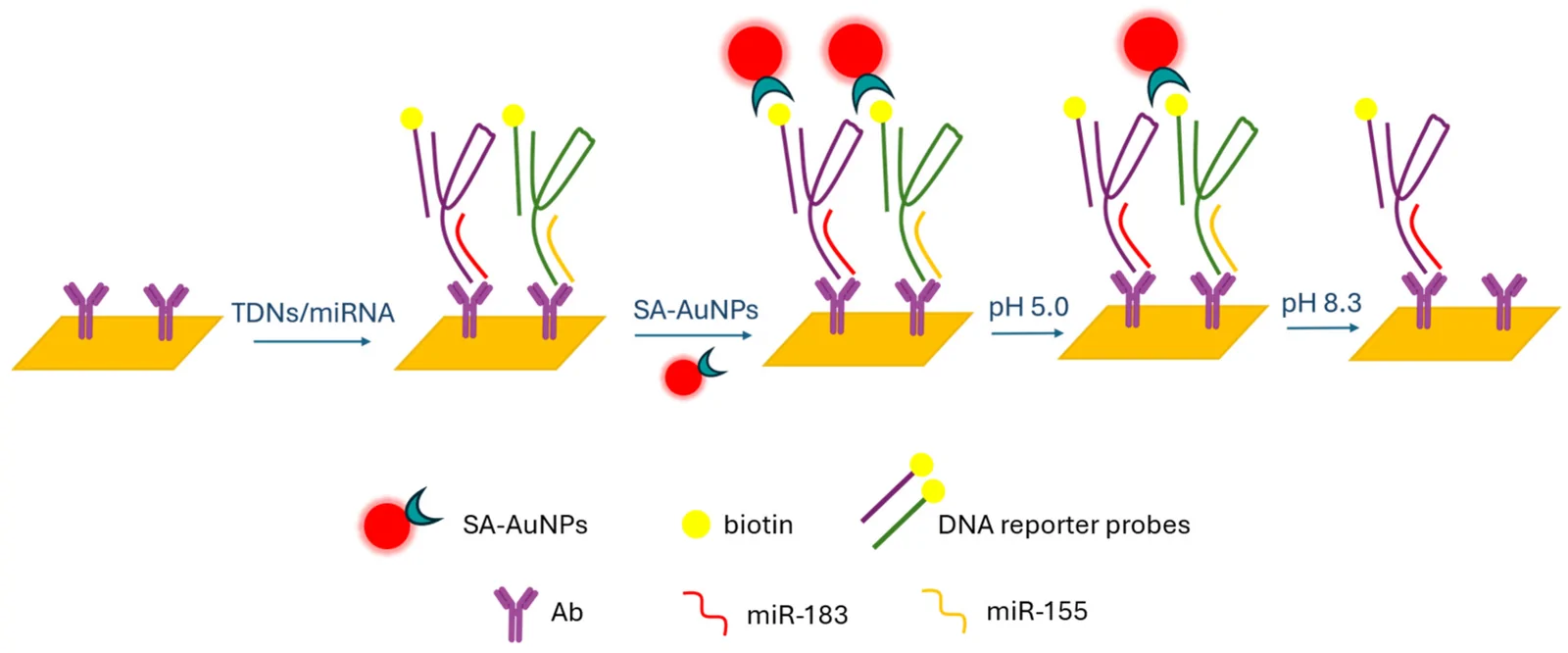
A multiplexed SPR sensor comprised by two pH-responsive DNA nanoswitches. The target miRNA molecules form DNA-RNA hybrids with biotinylated DNA probes and are immobilized on the sensor’s surface via antibodies and labeled with AuNPs via biotin-streptavidin affinity. pH adjustments induce detachment of AuNP conjugates to produce changes in SPR signal. TDN: triplex DNA nanoswitch.

SPR sensors offer label-free detection of biomolecular interactions with high detectability and real-time monitoring. An SPR sensor was developed for the detection of miR-183 and miR-155 in urine. Two triplex DNA nanoswitches with distinct pH responsiveness, each one containing a biotinylated reporter probe, where used to capture microRNA molecules. The complex was introduced to the sensor and captured by an antibody that recognizes DNA/RNA hybrids and was immobilized onto the sensor’s chip surface. Streptavidin-gold nanoparticle (AuNPs) conjugates bound then to the biotinylated probe for SPR signal enhancement. Multiplex signal acquisition was then achieved by adjusting the pH of the reaction solution to induce the transformation of the triplets, that resulted in the detachment of the AuNP-conjugates from the reporter and therefore changes in SPR signal which was proportional to the miRNA concentration at specific pH value (Figure 2). The LODs of the sensor were 0.36 pM for miR-183 and 0.46 pM for miR-155 in the linear range of 1 pM to 10 nM at pH 5.0 and pH 8.3, respectively, for single assay and 0.57 pM and 0.83 pM, respectively, for dual detection. Finally, the sensing platform had good discrimination between patients with bladder cancer and healthy individuals [78]. A novel optical fiber sensor based on SPR was also developed for the noninvasive detection of the bladder cancer biomarker NMP22 with real-time monitoring. A specific to NMP22 antibody was immobilized onto the optical fiber through a gold film coated onto the sensor’s surface and upon binding of the target protein, changes in the refractive index were recorded that resulted in a red shift in the SPR peak. A linear response range was determined in the range of 0–25 ng/mL with an LOD of 0.092 ng/mL, AUC of 0.8961 and recoveries ranging from 100.31% to 108.35%. From the analysis of real samples for patients and healthy people, using a pre-set threshold of 2.2 ng/mL, the sensor achieved sensitivity and specificity of 81.8% and 76.7% respectively, and compared to the same values using an ELISA test that were 87% and 73.3%, respectively. The classes were moderately unbalanced in a cohort of *n* = 38 with 23 urine samples from BC patients and 15 healthy urine samples [79].

### 2.5. Other Optical Sensors

A dual optical microfiber sensor was developed for the simultaneous detection of PSA proteins and long noncoding RNA prostate cancer antigen 3 (lncRNA PCA3). The sensor was constituted from two microfibers decorated with gold nanorods for PSA and gold nanobipyramids for PCA3 with strong localized surface plasmon resonance (LSPR) effects. The binding of the targets induced a refractive index change resulting in a measurable wavelength redshift. Results showed LODs of 3.97 × 10^−15^ mol/L for PSA and 1.56 × 10^−14^ mol/L for PCA3 in buffer, values that are three orders of magnitude lower than those of existing state-of-the-art technologies for PCa detection. The system allowed for real-time monitoring of optical spectral variations and was applied to undiluted clinical human serum and urine samples [80]. Liquid crystal-filled transmission electron microscopy in combination with the cationic surfactant octyltrimethylammonium bromide (OTAB) and double-stranded DNA (dsDNA) was also used for highly specific and label-free detection of platelet-derived growth factor (PDGF-BB). Elevated urinary PDGF-BB levels have been associated with prostate cancer and proposed as a potential non-invasive biomarker for early cancer detection [81]. The principle of the method involved adsorption of OTAB-coated 4-cyano-4′-pentylbiphenyl (5CB) on the TEM grid and dsDNA. Upon addition of the target PDGF-BB, a homeotropic-to-planar configurational change in 5CB was induced, which was observed by polarized optical microscopy. The method yielded an LOD of 2.63 pg/mL within a range of 5–200 pg/mL with a total analysis time of 60 min. The method was finally applied to human serum and synthetic urine samples [82]. Another tapered optical fiber sensor was developed for the detection of hCG. The fiber was decorated with AuNPs. Upon binding of the target, a shift in wavelength was measured. After spiking the target into urine, the sensor achieved a LOD of 0.03 mIU/mL [83].

### 2.6. Electrochemical Sensors

Electrochemical sensors are one of the most significant analytical tools for liquid biopsy applications, because of their simplicity, user-friendliness, high detectability/sensitivity, excellent specificity, rapid response, rapid analysis and cost-effectiveness. They also do not require complicated sample pre-processing even in complex matrices. The use of novel nanomaterials, like MOFs and GO, other nanostructures with high porosity and large surface area with outstanding electrical conductivity, metallic nanoparticles and flexible materials enhanced the analytical performance and the cost-effective fabrication of these sensors. Also, electrochemical sensors allow for miniaturization, re-usability and label-free detection. Label-free detection is accomplished by using redox probes, such as ferrocene, methylene blue, Prussian blue and thionine that enable the re-usability of the electrochemical sensors and increase their simplicity [9,84,85]. Among others, paper-based electrochemical devices are ideal for POCT and low-resource settings, because they are portable, disposable, cost-effective, can be mass-produced, provide rapid processing and require minimal sample volume [86].

#### 2.6.1. Metabolites, Small Molecules, and Ions

This category of electrochemical platforms targets urinary metabolites, small molecules, and ionic biomarkers that are associated with cancer-related changes in metabolism. Since some of these analytes allow direct monitoring of aberrant biochemical pathways and/or can be monitored without the use of complicated biorecognition structures, they are generally easier to monitor than proteins. The majority of recent studies regarding this area have primarily focused on improving catalytic rates, electron-transfer rates, operational stability, and point-of-care compatibility via nanostructure conductive interfaces, molecular imprinting, wearable electronic devices, and machine-learning-assisted data analysis.

One of the most well-studied urinary metabolites for detecting PCa continues to be sarcosine (SAR). Both enzyme-based and non-enzyme-based sensing strategies have shown great analytical sensitivity. Xu et al. 2026 created a self-powered, dual-mode wearable and enzyme biofuel cell electrochemical biosensor for SAR detection. Enzyme biofuel cell-based sensors are very promising for wearable sensors due to their self-powered potential. These sensors can have external power-free operation, because they convert chemical energy into electrical energy through enzymatic catalytic reactions, enabling self-powered detection of analytes. Urine can be used for such systems, as endogenous substances in urine can be effectively utilized as fuel to provide continuous self-energy for sensing. In this reported sensor, SAR was oxidized by SOx immobilized on hollow CuSe electrodes. A zinc sheet was used as a cathode, while Zn^2+^ in urine served as the electrolyte and SAR as the biofuel for the zinc-based self-battery. Upon SAR oxidation, which was monitored with CV, H_2_O_2_ was produced, which subsequently oxidized TMB in the presence of HRP. The sensor allowed for both electrochemical (via biofuel cell) and visual (via TMB colorimetry) detection. The LOD for electrochemical detection was 0.85 pM within the range of 10 pM–500 μM, while for colorimetric detection the LOD was 1.14 pM. The dual-mode sensor also exhibited RSDs < 5%. Integration with a hybrid classification-regression model enhanced quantitative accuracy with a correlation between true and predicted values of R^2^ = 0.980. A Bluetooth accessory also provided wireless real-time monitoring through a smartphone application for colorimetric detection, but the detectability was not as good with an LOD of 0.15 μM [7]. Simultaneous detection of SAR and creatinine in urine was accomplished through a potential switched dual-mode nCuCd bioelectrode developed by Stasyuk et al. 2026. A nCuCd-MOF was synthesized and demonstrated peroxidase-like nanozyme activity and a coordination-active surface for N-methylhydantoin, utilized in SOx-initiated H_2_O_2_ reactions and creatinine deiminase catalyzed reactions, respectively. The sensor achieved an LOD of 0.35 μM for SAR over a range of 1–14 μM and an LOD of 0.20 μM for creatinine across a range of 5–105 μM and RSDs < 5% [87]. Rasheed et al. 2025 developed a non-enzyme-based MOF derived graphitic carbon/NiVO_4_ composite that had a mesoporous honeycomb structure with high porosity for the non-enzymatic detection of SAR on a glassy carbon electrode (GCE). Graphitic carbon enhanced the surface area and thus the electrochemical performance of the sensor, while vanadium increased the number of interaction sites with SAR. The enhanced catalytic rate and electron-transfer properties of the material allowed for detection of SAR at a concentration as low as 0.03 µM. The standard addition method was used to minimize matrix effects and increase accuracy. The sensor achieved good urine recoveries in the range of 95–105% and good operational stability for up to 100 cycles [8]. Hamdi et al. 2025 further improved their ability to recognize SAR using a combination of aptamer-sensing and molecular-imprinting techniques on an adjusted by AuNPs/GCE and covalent organic framework@carbon nanofiber (COF@CNF) nanocomposite. The synergistic dual-recognition binding mechanism enabled sub-picomolar impedimetric detection. Dynamic range and LOD were 500 fM–50 pM, 50 pM–350 pM, and 0.166 pM for CV and EIS, respectively. Furthermore, the platform exhibited very high selectivity against structural analogues of amino acids [18]. Yao et al. 2026 developed a SAR sensor based on a molecularly imprinted hydrogel adhered to a piezoelectric strain gauge, which transduces SAR-induced hydrogel swelling into electrical resistance changes. Various hydrogel fabrication and interfacial coupling methods were explored. The optimal performance in respect to stability and reproducibility was demonstrated by polymerization of acrylamide, bisacrylamide, 4-vinylpyridine, and 2-acrylamido-2-methylpropanesulfonic acid. During polymerization, SAR added to the reaction mixture interacted through hydrogen bonding and electrostatic interactions with the monomers. SAR was subsequently removed to form the molecularly imprinted cavities. The hydrogel was coupled to the strain gauge through molecular bridging employing 3-(methacryloyloxy)propyltrimethoxysilane for covalent linkage. The sensor exhibited a dynamic range of 10^−3^–10^6^ μM and a response time of 2 min. In addition, this transduction strategy addressed matrix interference and enabled analysis without sample pre-treatment [88].

In addition to the investigation of PCa-specific urinary metabolites, several additional metabolites/ions associated with PCa have been studied using various sensing methodologies including label-free and molecular imprinting approaches. Dehghani et al. 2024 developed an impedance-based label-free sensing platform (non-faradaic) for measuring urinary Zn^2+^ (UZn), an excellent biomarker for PCa. Normal prostate tissue reveals a large accumulation of zinc, which leads to a 10-fold higher concentration compared with other soft tissues, that is decreased even at early stages of cancer. The principle of the sensor involves a screen-printed carbon electrode (SPCE) modified with a porous composite of cellulose acetate/reduced graphene oxide composite with increased porosity for enhanced UZn capture. Zn^2+^-induced changes in the electrical double layer resulted in significant modulation of the measured impedance allowing for quantitative measurement over a large dynamic range (0.1–1000 ng/mL) with an LOD of 7.33 ng/mL in artificial urine and good correlation with ICP-MS results for clinical urine samples [89]. Subsequently, the authors described a molecularly imprinted poly(phenol sulfonic) (PPS) nanofilm-integrated extended gate field effect transistor for detection of spermine. Reduced urinary spermine concentrations have been associated with prostate cancer and investigated as a non-invasive biomarker for cancer detection such as prostate and urogenital malignancies [90]. By utilizing both extended gate field effect transistor (EGFET) and differential pulse voltammetry (DPV) transduction mechanisms, in combination with MIP, the platform achieved an enhanced detection limit of 1.23 ng/mL, and a dynamic range from 0.1 to 1000 ng/mL in artificial urine with IF ≈ 4.1. The detectability was 10 times higher than non-imprinted polymers, and the sensor showed excellent correlation with LC-MS analysis of clinical urine samples. These studies exemplify the increasing trend towards developing miniaturizable and reusable point-of-care electrochemical platforms capable of measuring PCa-specific metabolites [19].

Moreover, several studies were directed towards investigating cancer-specific metabolites related to neuroendocrine tumors and oxidative stress pathways. Urinary 5-hydroxy-indoleacetic acid (5-HIAA) is an established biomarker used for the diagnosis and monitoring of serotonin-producing neuroendocrine tumors, particularly those associated with carcinoid syndrome [91]. Demkiv et al. 2025 developed amperometric sensors for detecting 5-HIAA, which is significant for carcinoid tumors, using laccase-mimicking Pt/Mn and MnO_2_ nanozyme materials. These materials possessed significantly better operational stability compared to native laccase, provided linear ranges of 0.3–15 μM and 2–50 μM and detection limits of 0.16 and 1.4 μM, respectively. Finally, the sensors exhibited comparable correlation with HPLC results for clinical urine samples with recoveries around 94.1% and RSDs < 5% [84]. Similarly, Soliman et al. 2025 developed a nickel monoligand–metal–organic framework (NiMLMOF) sensor for detecting homovanillic acid, a tumor biomarker for neuroblastoma and other dopamine-related disorders. Electrochemical performance was evaluated both by EIS and CV, while calibration graphs were constructed by DPV. Enhanced catalytic oxidation of the analyte compared to bare electrode and analyte pre-concentration effects facilitated simple and rapid urine analysis with good selectivity when compared to traditional chromatographic methods. The modification of the electrode improved the detectability and electrocatalytic efficiency of the sensor, providing recoveries of 93.2–97.6%, RSDs < 2%, a dynamic range from 0.1 μM to 70.0 μM and an LOD of 0.08 μM in buffer [92].

Overall, electrochemical sensing of urinary metabolites has transitioned toward portable, multifunctional, and miniaturized systems that utilize catalytic nanomaterials, molecular recognition elements, and computational analysis tools. Several systems showed excellent analytical sensitivity and provided satisfactory analytical performance within complex urine matrices. However, numerous studies continue to lack sufficient clinical validation and long-term testing under realistic operating environments.

#### 2.6.2. Proteins and Antigens

Electrochemical protein-based biosensors are among the most researched tools for non-invasive urinary cancer diagnosis, because of their ability to detect both circulating protein biomarkers and tumor-associated antigens. The majority of these biosensors utilized antibodies or aptamers as biorecognition molecules in combination with nanostructured conductive surfaces to increase electron transfer efficiency and/or increase the number of bioreceptors that can be loaded onto each surface. Impedance, current suppression at specific potentials (voltammograms) and changes in electrochemical signals produced when biomolecules interact with the electrode’s surface, have primarily been used to generate signal transduction [9,86,93,94].

In addition to several studies evaluating bladder and prostate cancers biomarkers, many research groups have reported label-free detection schemes utilizing highly conductive nanostructures as transducers targeting proteins and antigens. For example, Sana et al. 2024 developed a paper-based electrochemical aptasensor for detecting the Epidermal Growth Factor Receptor (EGFR), a biomarker for uterine cancer, utilizing silver nanowires (AgNWs)-modified carbon electrodes. The binding of the target to the immobilized aptamer reduced the current flow at the working electrode in the presence of the redox pair 0.5 mM potassium ferro/ferri cyanide that was monitored by CV within 30 s. The sensor resulted in a dynamic range of 0.01 ng/mL–1 μg/mL with a detection limit of 0.01 ng/mL and good recovery results (94.8–97.4%) in urine samples. This sensor is inexpensive, disposable, easy to use, and with very good repeatability [86]. Other studies have shown that EGFR can be used as a potential prognostic biomarker in urothelial bladder carcinoma [95]. Another group developed a ratiometric immunoassay for the bladder cancer biomarker nuclear matrix protein 22 (NMP22) detection on a ZIF-8@MWCNTs@Chit@Fc@AuNPs composite. NMP22 is one of the most important tumor markers of bladder cancer. The researchers introduced novel nanomaterials for electrode modification to overcome the limitations of low reproducibility. They simultaneously modulated methylene blue and ferrocene (Fc) signals and utilized MOFs (ZIF-8) with multi-walled carbon nanotubes (MWCNTs) and chitosan (Chit) to increase the surface area and the conducting properties to obtain better analytical robustness (accuracy and repeatability/reproducibility) minimizing external interferences and lowering detection levels in the fg/mL-range (3.33 fg/mL) with an extremely wide dynamic range of 0.01 pg/mL–1000 ng/mL and great reproducibility (RSD = 2.23%) and selectivity [9]. Similarly, Junlapak et al. 2026 reported a label-free NMP22 immunosensor using a SPCE modified with carbon decorated with gold nanoparticles (AuNPs@OMC) and carboxylate graphene-supported thionine (Thi@Gr-COOH) nanocomposites. Thionine was reduced and differences in peak current were monitored by DPV upon capturing of the target to immobilized antibodies onto the nanocomposites to obtain an ultra-low detection limit of 2.96 × 10^−8^ ng/mL with a dynamic range of 1.0 × 10^−7^ to 1.0 × 10^−1^ ng/mL, good selectivity, good validation against the commercially available NMP22 BladderChek assay, recoveries of 80.1–103.8% and RSDs of 0.1–4.7% in urine. The combination of the above nanostructures resulted in 2.25 times higher detectability, while the signal from urine interferences was <5% [85]. He et al. 2026 further increased the specificity of NMP22 detection via an epitope-imprinted electrochemical biosensor (EMIP) fabricated on thiolated graphene/AuNP-modified gold electrodes. The synergetic effect of graphene and AuNPs, providing high conductivity and an increased number of binding sites, was used to enhance the detectability of the sensor, while EMIP fabrication, utilizing a short peptide segment of the whole protein as the template, reduced the cost of the sensor. The sensor provided a wide linear range of 0.1 pg/mL–1 µg/mL with a detection limit of 0.077 pg/mL and relatively fast assay times of about 20 min. Application of the sensor to human urine samples showed recovery rates ranging from 96.92% to 100.88% [96].

**Figure 3:**
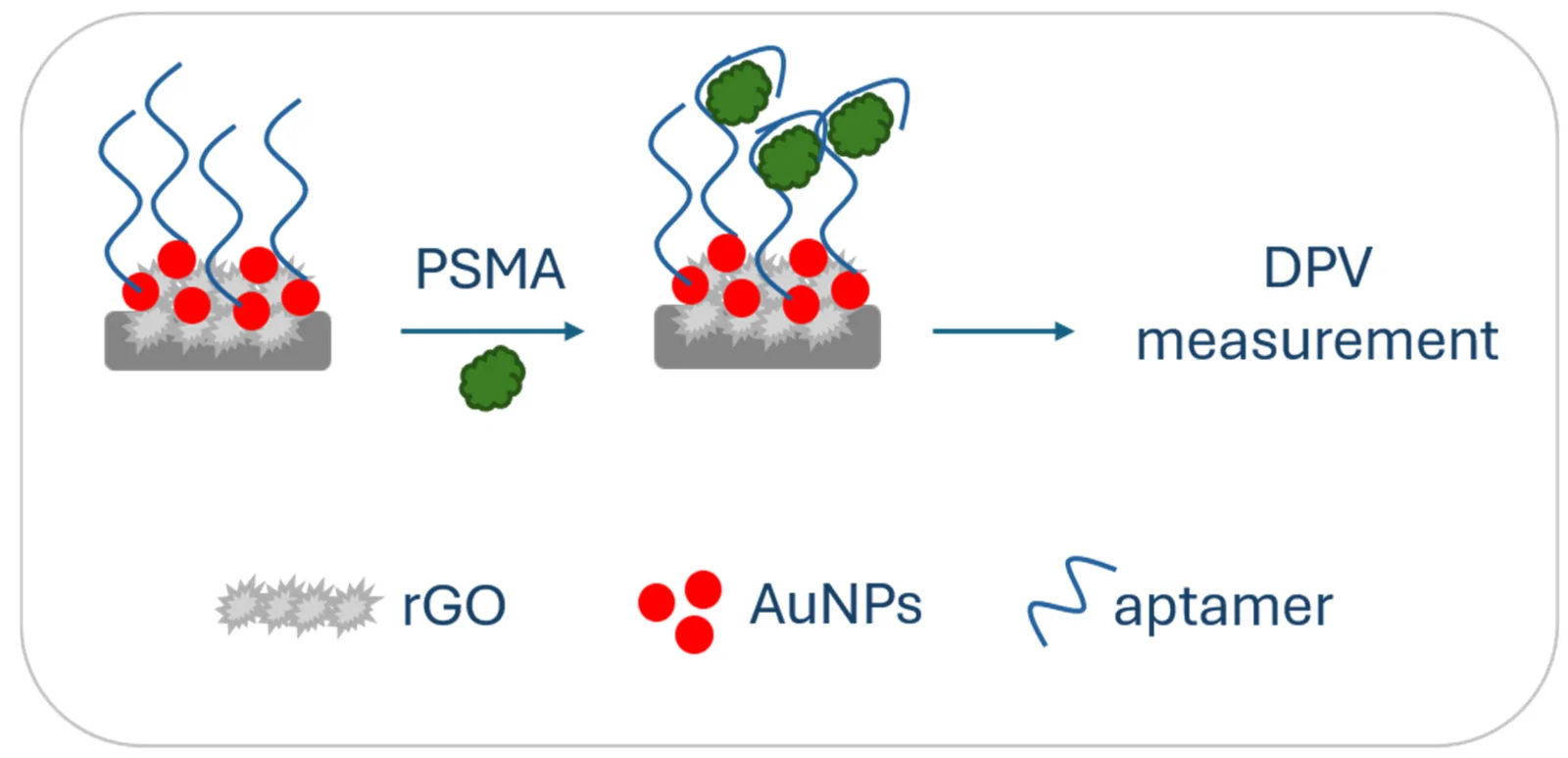
A 3D-rGO/AuNP multiplexed aptasensor for urinary prostate-specific membrane antigen (PSMA) detection. The measurement is performed using a portable reader on a three-electrode architecture where specific PSMA aptamers are immobilized through deposited reduced graphene oxide (rGO) and gold nanoparticles (AuNPs) for PSMA binding.

PCa-associated proteins were also extensively investigated through nanomaterial-enhanced immunosensing and aptasensing strategies. Li et al. 2025 designed a photoelectrochemical (PEC) immunosensor for PSA using MoS_2_/N,S-CQDs/Bi_2_S_3_ nanoheterojunction deposited on fluorine-doped tin oxide (FTO) electrodes. Highly sensitive photocurrent response was obtained by effective separation of electrons/holes under illumination with visible light minimizing background interferences. The linear range was 0.001–50 ng/mL and LOD 0.35 pg/mL, RSD < 1.54% and spiked recoveries ranged from 97.4% to 103.0% [93]. A similar application was reported by Rizwan et al. 2026 with label-free disposable nano-immunochips for PSA detection in urine and serum using rGO modification of SPE. The chips provided an exceptionally broad detection range from 0.10 fg/mL to 100 ng/mL, ultralow fg/mL (0.10 fg/mL) detectability, with recoveries of 97–107% and RSDs of 4.02–4.57% in spiked artificial urine samples, and good validation using clinical oncology urine samples [94]. An ultrasensitive cascade enzyme immunoassay microfluidic chip was developed by Ai et al. 2026 for simultaneous detection of PSA and Engrailed-2, a transcription factor that serves as a new biomarker for PCa. A Fe_3_O_4_ magnetic MOF was functionalized with primary antibodies specific to each analyte. Antibodies conjugated with glucose oxidase bound to a different epitope of the analyte to form a sandwich complex. The immunocomplexes were directed to the microfluidic channel by an external magnetic field. Glucose oxidase oxidized glucose to produce H_2_O_2_ and a Co_3_O_4_ MXene-modified electrode was used for amperometric quantification. The microfluidic chip achieved an LOD of 0.154 pg/mL for PSA and 0.146 pg/mL for Engrailed-2 over a linear range of 0.001–100 ng/mL, recoveries of 97.3–106.5%, and RSDs < 9.01% [97]. Moreover, Amin-Sadrabadi et al. 2026 developed a 3D-rGO/AuNP multiplexed aptasensor for urinary prostate-specific membrane antigen (PSMA) detection. The measurement was performed using a three-electrode architecture for enhanced electron transfer that allowed simultaneous replicate measurements (Figure 3). The sensor exhibited an LOD of 5 pg/mL, a linear range of 15–2000 pg/mL, >30 min for analysis of real samples, and RSDs ≤5% and improved the analytical reproducibility for point-of-care prostate cancer diagnostics [98]. Other applications for PCa biomarkers include (i) an APTES-coated Fe_3_O_4_/GO magnetic nanoparticle-based immunosensor for Engrailed-2 (EN2) detection developed by Yașar et al. 2026 for robust immobilization of antibodies to improve the reproducibility that exhibited selective detection within the range of 75–150 nM with an LOD of 41 nM and an AUC value of 0.65 [99] and (ii) a low-cost and user-friendly pencil graphite electrode-based impedimetric immunosensor for label-free detection of carcinoembryonic antigen (CEA) that is abnormally expressed in many cancer types. This sensor demonstrated an LOD of 4.4 pg/mL, a wide linear range from 13.2 to 1 × 10^5^ pg/mL, real-time monitoring, high reproducibility and specificity and recoveries in the range of 86–94% in artificial urine samples [100].

Other studies have evaluated larger sets of oncologic proteins and have implemented multiple-detection formats. Parambil et al. 2025 developed a multi-component MoS_2_/gC_3_N_4_/carbon dots nanohybrids immunosensor for label-free detection of human chorionic gonadotropin (hCG), a biomarker for bladder, ovarian, and prostate cancers. Hormone hCG and related molecular forms, including free β-hCG and the β-core fragment, have been investigated as tumor markers in several malignancies. As the relative abundance and diagnostic relevance of different hCG molecular forms may vary according to tumor type, considering these forms alongside intact hCG provides a more accurate representation of its role as a cancer biomarker. The sensor achieved a linear range of 10^−4^–80 ng/mL with a detection limit of 0.015 ng/mL and recovery of 99.5% in spiked urine samples and significantly reduced assay time to about 3 min as compared to traditional ELISA assays [101]. Finally, Arefian et al. 2026 developed an Ag@ZIF-90-based immunosensor for the detection of the pancreatic cancer biomarker carbohydrate antigen 19–9 (CA19-9). The sensor displayed an ultrasensitive-wide detection range from 10^−4^ to 2000 U/mL, an LOD of 9 × 10^−5^ U/mL, excellent specificity, good reproducibility with RSDs < 4.6%, recoveries of >97.2%, an extraordinary long-term stability up to 40 days and strong agreement with the results from ELISA [3].

Beyond conventional immunosensing approaches, enzyme activity-based electrochemical strategies have also been explored for indirect monitoring of tumor-associated enzymes. Wang et al. 2025 developed an ultrasensitive electrochemical urine-biopsy platform based on a tandem peptide–peptide nucleic acid (PNA) artificial biomarker system for the detection of Cathepsin B activity. Following tumor-specific proteolytic cleavage, released PNA reporters were quantified using a Nb.BbvCI-assisted DNA-walker biosensor, where cyclic cleavage amplification generated square wave voltammetry (SWV) signals with a detection limit of 17.5 pg/mL. The nuclease-resistant PNA architecture improved stability under physiological conditions, while the integration of fluorescence imaging and electrochemical quantification highlighted the growing convergence of synthetic biomarker engineering and multimodal cancer diagnostics [102].

Overall, electrochemical protein biosensors demonstrated remarkable analytical sensitivity, broad dynamic ranges, and increased integration with portable and AI-assisted diagnostic platforms that are discussed below. Nanostructured conductive scaffolds, ratiometric sensing strategies, molecular imprinting, and multiplexed electrode configurations substantially improved assay detectability, robustness and reproducibility due to the enhanced electron transfer and increased number of binding sites of biorecognition molecules and targets. Nevertheless, despite promising analytical performance, many studies remain limited by relatively small validation cohorts, incomplete long-term clinical studies, and the need for simplified sample-processing workflows suitable for routine POCT.

#### 2.6.3. Nucleic Acids

Electrochemical nucleic-acid-based biosensors exploit the high sequence specificity of RNA, miRNA, and DNA hybridization to enable ultrasensitive detection of cancer-associated genetic biomarkers in urine. Compared to protein-based assays, electrochemical nucleic acid-based assays provide higher specificity and may be more valuable for early-stage cancer detection, as circulating nucleic acids in body fluids can be detected even in trace amounts. Recently, the use of various combinations of aptamer-based biorecognition, nanostructured conductive interface materials, isothermal amplification strategies, and signal amplification techniques has led to significant improvements in the detectability and overall analytical performance of these types of biosensors. As many of these types of electrochemical biosensors can now be constructed into miniaturized formats, they can also be promising for POCT.

**Figure 4:**
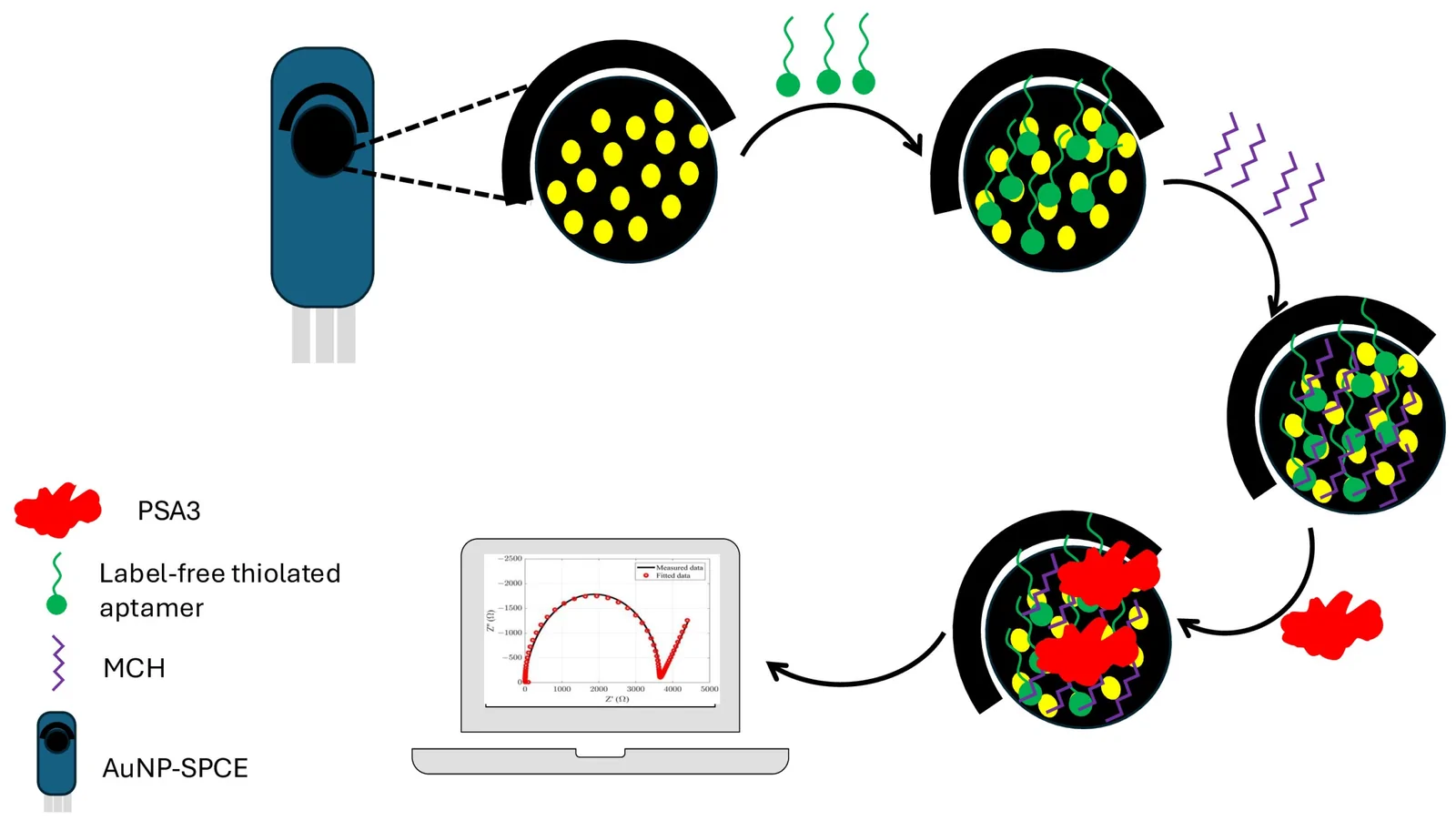
A label-free impedimetric aptasensor based on CG3 RNA aptamers attached to AuNP-modified screen-printed carbon electrodes (SPCEs) for prostate cancer antigen 3 (PCA3) detection. The working electrode with AuNPs is modified with label-free thiolated aptamers and its surface is blocked with MCH. The sensor is based on target binding-induced conformational changes resulting in the reduction in the current of a ferri/ferrocyanide redox probe monitoring by electrochemical impedance spectroscopy (EIS). MCH: 6-mercapto-1-hexanol.

PCA3 is one of the most extensively investigated urinary RNA targets evaluated for PCa using electrochemical nucleic acid-based biosensors. A label-free impedimetric aptasensor based on CG3 RNA aptamers attached to AuNP-modified SPCEs for signal enhancement was recently reported by Takita et al. 2024. This sensor utilized PCA3 binding-induced conformational changes and sterically hindered electron flow from ferri/ferrocyanide redox probe that resulted in concentration-dependent increases in charge-transfer resistances measured by EIS (Figure 4). The reported LOD was 1 fM in buffer and 20 fM in artificial urine over a wide linear range (0.1 pM–1 nM), while demonstrating high selectivity against non-complementary sequences and protein interferences [103]. Similar results were also obtained by Mokni et al. 2024. The authors published a label-free electrochemical genosensor that consisted of thiolated-DNA probes immobilized on gold electrodes, avoiding the need for a redox probe. The method involves total RNA isolation from urine samples, RT-PCR for PCA3 gene amplification and hybridization onto the electrode’s surface through the ssDNA probes after denaturation of the amplified sequences. Hybridization of PCA3-RNA fragments caused suppression of the electrochemical reduction signal of nucleobases. Detection was performed via CV and SWV. The LOD achieved was 1.6 ng/mL using the optimal DNA probe over a linear range of 0.1–1 μg/mL [104].

**Figure 5:**
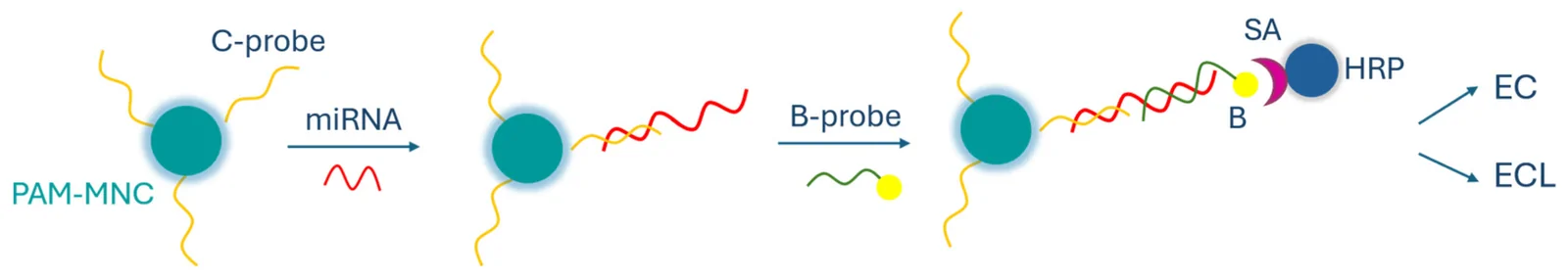
A dual-mode electrochemical/electrochemiluminescent (ECL) biosensor for miRNA-183-5p detection. A sandwich-hybridization-assay, between a capture and a detection DNA probe and the target is performed on a polyacrylamide-grafted magnetic-nano-composite (PAM-MNC) platform. Detection is based on horseradish peroxidase (HRP) and luminol for electrochemical (EC) and ECL signal. C-probe: capture probe, B-probe: biotinylated detection probe, SA: streptavidin, B: biotin.

Numerous reports described sensitive miRNA-detection using hybrid amplification and/or nanomaterial-assisted transduction approaches. An example of such a report is the silver-NP mediated-sandwich hybridization sensor for urinary miR-21 detection for PCa diagnosis presented by Pang et al. 2024. Target miRNA was hybridized to a capture DNA probe that carried a biotin at one end and a detection probe which carried a NH_2_-group at 5′ end. The complex was captured onto streptavidin-modified SPCEs and subsequently silver nanoparticles (AgNPs) interacted with the amino groups for signal generation. The AgNP-amplified signal allowed for fast SWV measurements that provided an extremely low LOD value of 100 fM. The method was applied to 38 clinical urine samples from patients with PCa, prostate hyperplasia and healthy donors, after microRNA isolation using an extraction kit. Finally, this sensor showed increased multiplexing potential of the simultaneous measurement of multiple miRNAs [105]. Another example is the interdigitated field-effect transistor (FET) containing metal-carbide/carbon-nanotube composites for exosomal miRNA-122 detection in urine, which is correlated with liver cancer, as presented by Zhang et al. 2025. Target miRNA was captured on the sensor’s surface by immobilized specific aptamers, while the electric-double-layer effect, along with the highly conductive MC@CNT material architecture, significantly amplified the signal output allowing for sensitive detection and quantitative determination of exosomal miR-122 in urine across a wide dynamic range of 1 fM–10 nM with an LOD of 0.12 fM. Results were consistent with those obtained by qPCR in clinical liver-cancer samples, while ROC analysis exhibited an AUC of 0.9776, a sensitivity of 0.92, and a specificity of 0.96, demonstrating effective differentiation between healthy individuals and liver cancer patients. Moreover, the platform is portable and includes a Wi-Fi module, enabling wireless signal transmission, recording, and storage [106]. Additionally, Fayisa et al. 2026 reported a dual-mode electrochemical/electrochemiluminescent (ECL) biosensor for miRNA-183-5p detection employing a polyacrylamide-grafted magnetic-nano-composite platform. ECL is attractive because it does not require an external optical source for excitation, simplifying the device and minimizing the background. The sandwich-hybridization-based, between a capture and a detection DNA probe, assay employed HRP-assisted signal amplification to simultaneously produce electrochemical and luminol-based ECL signals from the same recognition event (Figure 5). LOD values were as low as 4.6–9.9 aM for EC detection and 4.1–5.1 aM for the ECL system in urine, serum and PBS matrices and linear responses in the range of 0.01–1000 aM [107].

Electrochemical and optical nucleic acid analysis in miniaturized formats and portable multi-analyte platforms were further improved through the integration of amplification-based diagnostic techniques. Koo et al. 2026 reported on the Accelerated non-inVasive bioAnalyte testing And Reporting (AVATAR) system for decentralized electrochemical profiling of a six-urinary-RNA-biomarker panel for PCa detection with instantaneous result reporting. The AVATAR system employed isothermal recombinase polymerase amplification (RPA) after RNA isolation and chronoamperometric read-out on specific primer-functionalized magnetic beads in combination with a wireless mobile module onto a portable electrochemical readout platform and a smartphone to enable decentralization. Biotin-modified uracil bases were incorporated into the newly synthesized amplicon strands during RPA, whereas the amplicons were detected through streptavidin-HRP conjugates and TMB oxidation onto SPEs. With this sensor, six genes; T2, ERG, PCA3, SChLAP1, HOXC6, DLX1, and KLK2 were quantitated in approximately 55 min using a single reaction mixture per RNA, with a limit of quantitation (LOQ) of 100 copies across a 10^2^–10^5^-copy linear range. Importantly, the AVATAR system showed good performance in clinical trials (*n* = 124), coefficients of variation (CVs) <8.9% and excellent correlation with comprehensive transcriptomic sequencing of matched tissue specimens and qRT-PCR. Finally, it demonstrated high discrimination ability of high-risk PCa cases with AUC values of 0.88 that makes this platform a promising candidate for clinical routine application [108].

In summary, electrochemical nucleic acid-based biosensors demonstrate very low LOD values, high specificity, portability and simplicity, and increased compatibility with decentralized testing systems. Further advances in nanostructured transducers, amplification-free sensing strategies, hybrid electrochemical–optical systems and portable multi-analyte devices will further improve analytical performance and clinical utility. However, additional clinical validation studies, standardization of assays and simplification of sample-preparation protocols are required prior to widespread clinical implementation of these biosensing systems.

#### 2.6.4. Extracellular Vesicles

Electrochemical biosensors for the detection of extracellular vesicles have emerged as highly promising platforms for non-invasive cancer diagnostics, because extracellular vesicles preserve the molecular characteristics of their parental tumor cells. They also play an important role in intercellular communication and can be accessible in body fluids and urine. For the above reasons, EVs are considered very important biomarkers for cancer diagnosis. Reliable quantification of EVs in urine is still challenging due to their low abundance and lack of validated methods. Most electrochemical strategies rely on aptamer-based sensing of specific biomarkers present on the surface of EVs, such as PSMA and CD63, and/or utilize various forms of nanostructures of the conductive scaffold to increase analytical detectability and sensitivity, providing advantages in terms of faster measurements, smaller required sample volumes, miniaturization and portability compared to traditional methods such as flow cytometry and RT-qPCR [109].

**Figure 6:**
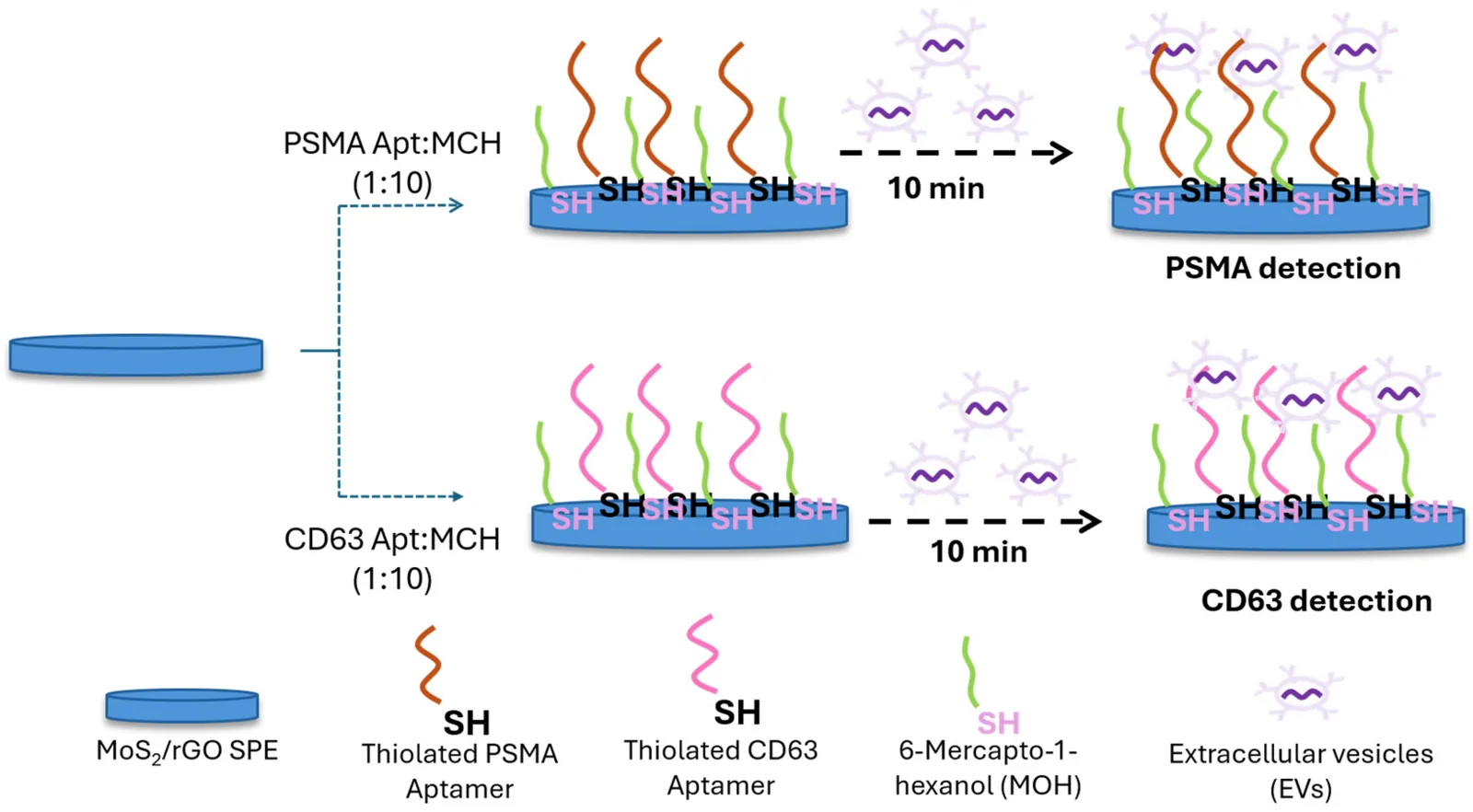
A label-free capacitive electrochemical biosensor for the detection of extracellular vesicles. Detection is based on the recognition of CD63 that is a generic marker present in all EVs and PSMA that comprises a prostate cancer biomarker through specific aptamers immobilized onto MoS_2_/rGO-modified screen-printed carbon electrodes (SPCEs). rGO: reduced graphene oxide.

In that context, Permpoka et al. 2025 introduced a label-free capacitive electrochemical biosensor capable of detecting PCa-associated EVs in urine, based on the targeting of CD63 that is a generic marker present in all EVs and PSMA that comprises a PCa biomarker. Specific aptamers were immobilized onto MoS_2_/rGO-modified SPCEs. The EV detection was based on changes in the non-faradaic capacitance generated when EVs bound to the electrode surface (Figure 6). The dual marker approach permitted normalization of the signal intensity with respect to the amount of EVs detected via aptamer binding to PSMA and the total number of EVs (normalized to creatinine) in each sample; thus, it greatly enhanced the reliability of the obtained results regardless of the variability of the samples. The required urine sample volume was 10 μL and the analysis was completed within 30 min. The capacitive sensor had a detection limit of 4.83 × 10^2^ EVs/μL and 1.47 × 10^3^ EVs/μL for PSMA and CD63, respectively, over a dynamic range of ~10^2^–10^5^ EVs/µL and with RSDs < 4.3% for PSMA and <10.5% for CD63. The results showed strong correlation with a commercial ELISA kit for PSMA, but with reduced total analysis time [109].

Similarly, Cao et al. 2025 presented a flexible electrochemical aptasensor for the direct detection of EVs by integrating silver/carbon conductive ink onto MOF-derived porous carbon nanosheets. In this system, CD63-specific aptamers immobilized on carboxyl-functionalized porous carbon nanosheets enabled selective EV capture, while the intrinsic Ag^+^/Ag^0^ redox system embedded within the conductive ink eliminated the need for external redox mediators. EV binding suppressed DPV responses through steric hindrance effects, enabling sensitive detection across a broad linear range of 10^2^–10^6^ particles/μL with an ultra-low detection limit of 29 particles/μL in approximately 80–90 min. The flexible nature of the sensor demonstrated its excellent reproducibility, resistance to interference from other substances, reliable recoveries (97–104%) and RSDs < 2.13% of spiked EVs into real urine and serum matrices [110]. Similarly, Amin-Sadrabadi and Omidfar 2026 combined magnetic separation with aptamer-recognition process to achieve ultra-sensitive and label-free detection of PSMA-positive EVs in urine related to PCa. Selective EV isolation was accomplished by using carboxyl-functionalized magnetic nanoparticles conjugated with CD63 aptamers, while electrochemical detection was performed on carbon nanofibers (CNF)-modified SPCEs. High electron transfer rates and efficiencies of immobilization of aptamers were achieved due to the hierarchical structure of carbon nanofibers, leading to a detection limit of 3 pg/mL in a concentration range from 10 to 1400 pg/mL, while the recoveries ranged from 91.8% to 105.4% with RSDs < 6%, indicating negligible matrix effects. Furthermore, the platform showed highly selectivity against non-prostatic, benign prostatic origins and PSMA-negative EV populations, high correlation with PSMA ELISA results, while urinary creatinine normalization improved data reliability [111].

In summary, electrochemical biosensors targeting EVs appear to be attractive tools for non-invasively identifying cancer through direct analysis of urinary tumor-exosome content. As has been previously discussed, advances in aptamer design, magnetic separation, nanostructure modification of novel conductive substrates with outstanding properties and label-free electrochemical transduction significantly improved assay detectability and sensitivity, practicality and portability. However, larger-scale clinical validation studies, standardization of procedures for isolating EVs, and improved inter-laboratory reproducibility are still critical barriers to future clinical applications.

#### 2.6.5. Volatile Organic Compounds and Multisensory Platforms

The use of electrochemical, volatilomic, and multicomponent analytical systems has shown great promise for non-invasive cancer diagnosis through their ability to detect unique biochemical “signatures” in bodily fluids such as urine and breath. Multisensory electrochemical and volatilomics platforms utilize a combination of chemical sensors (i.e., partially selective) with chemometric and machine learning techniques to differentiate metabolic profiling of patients from those of healthy individuals. In comparison to traditional single analyte biosensors, these systems offer broader metabolic coverage, rapid analysis times, and compatibility with portable point-of-care screening.

Independent volatile profiling techniques have also been investigated through gas-sensor array technologies. It is well established that cancer cells exhibit different metabolic profiles from those of normal tissues. The analysis of volatile organic compounds (VOCs), which are small metabolites, has emerged as a promising approach for cancer screening and disease monitoring. VOCs are continuously produced within the human body and released through exhaled breath, blood, skin, urine, and feces, thereby providing valuable information about the physiological and metabolic state. However, the development of reliable and effective analytical methods for VOC detection remains one of the major challenges in cancer research [14]. Arasaradnam et al. 2025 developed a urinary VOC analytical platform that consisted of solid phase microextraction in association with an electronic nose (eNose) constructed of eight metal oxide sensors for colorectal cancer screening. Resistance patterns obtained from VOCs were analyzed using feature extraction, principal component analysis, and radial basis function neural network classification to enable discrimination between colorectal cancer patients and healthy individuals with an AUC value of 0.88. The neural network was trained using five-fold cross-validation on a cohort from the study FAMISHED (*n* = 22 healthy control samples, *n* = 53 colorectal cancer patient samples) and tested in an independent cohort from the study RECEDE (*n* = 1681 total samples, *n* = 763 negative controls). Because the classes were imbalanced, a precision–recall curve was calculated to provide a reliable measure of the sensor’s performance. The platform showed excellent multiplexing capabilities by allowing simultaneous profiling of broad-spectrum cancer-associated volatile signatures while providing high-throughput capability for clinical analyses [112]. Electrochemical multi-signal platforms have also been investigated for monitoring enzymatic activity related to cancer progression. A hyaluronidase-driven platform integrated with an enzyme-degradable hyaluronic acid-hydrogel that contained magnetic beads with periodic magnetic-modulated electrochemical analysis was reported. Hyaluronidase is a promising urinary biomarker for bladder cancer diagnosis without sample pre-treatment requirements. When hyaluronidase catalyzed the degradation of the hydrogel, magnetic beads were released. The beads were functionalized with electrochemically active substances on their surface and therefore were detected by a periodic magnetic-modulated electrochemical analysis system that generates chronoamperograms with alternating magnetic fields providing frequency domain signals free from baseline drift interferences. The system provided a linear range of 2–25 U/mL an LOD value of 0.414 U/mL, and strong selectivity for artificial hematuria samples. In addition, the specificity of hyaluronic acid-based hydrogel degradation, combined with the magnetic, minimized interferences from other urinary components [113].

Multisensory electrochemical VOC-based systems show potential for rapid highly multiplexed non-invasive cancer diagnostics through biochemical fingerprint analysis. Improvements in chemometrics processing/machine learning-based classification, sensor array engineering and multimodal data fusion, that are discussed below, provided significant improvement in diagnostic accuracy across diverse cancers. However, larger clinical cohorts and external validation studies are required for implementation of such sensors in clinical practice.

### 2.7. Photothermal Sensors

A high-throughput aptamer-based photothermal sensing platform has been developed using MnO_2_@CuS nanoprobes for the detection of bladder cancer cells. A specific aptamer targeting epithelial cell adhesion molecule (EpCAM) was immobilized on an amino-functionalized glass slide to capture the bladder cells, while a second aptamer was conjugated to MnO_2_@CuS nanoprobe that was used for the photothermal signal output. Upon 808 nm laser irradiation, infrared thermographic imaging was recorded to monitor the elevation of the temperature, enabling detection and quantitative determination of cell concentration. This platform showed a linear range of 10–10^6^ cells/mL with LOD and LOQ values of 2 and 7 cells/mL, respectively; recoveries of 98.2–102.0%; and RSDs of 1.7–3.2% [114].

### 2.8. Machine Learning-Assisted Sensors

Recently, artificial intelligence, including machine and deep learning algorithms, have been introduced in bioanalysis for signal and image processing and feature and pattern extraction from complicated data, such as spectra, to assist diagnosis and decision making and increase the accuracy of the interpreted results [74].

#### 2.8.1. Optical Sensors

A microfluidic dual-fluorescence and low-cost flow cytometry platform was developed for urinary cytology analysis. A phenotypic characteristic parameter was correlated to the size of the cell and nucleus and used for the differentiation of bladder cancer cells (tumor cells) and uroepithelial cells (normal cells) that was based on nuclear and cytoplasmic fluorescence signals from cells stained with Calcein AM and Hoechst 33342. Cell differentiation and data analysis were assisted by the development of a support vector machine (SVM) machine learning algorithm. The cells were split into two sets: a total of 70% of the cells were used for SVM training and 30% of the cells were used for cross-validation. Results demonstrated an accuracy of 84.7%, a specificity of 91.0% and a sensitivity of 75.0%. Validation using new test data sets of SV-HUC-1 cells (*n* = 6387) and 5637 cells (*n* = 3000) resulted in a false-positive rate of 10.19% for SV-HUC-1 cells and a false-negative rate of 20.53% for 5637 cells. The total analysis time includes a 15-min incubation time for staining, followed by microfluidic analysis and real-time data acquisition. The system was applied to exfoliated cells from patient urine samples (*n* = 6) diagnosed with bladder cancer and urinary tract infections [115]. An innovative urinary VOCs profiling platform featuring a colorimetric sensor array and deep-learning-assisted interpretation of results enabled the diagnosis of three types of cancer: bladder, colorectal, and prostate. Experiments were conducted using six VOCs as biomarkers for each cancer type. Sensing elements were exposed to a VOC-saturated environment for 12 h to elicit a colorimetric response. A library of combinations of 20 ionizable cationic dyes and 20 anionic dyes that displayed color changes in response to VOC exposure was used for high-throughput screening of candidate sensing elements. Dyes interacted with VOCs through Schiff base reactions, hydrogen bonding, acid-base interactions, redox reactions, and π–π stacking. Further screening of 17 promising candidate sensing elements, by varying concentration gradients, mixing ratios, and pH conditions, was performed using a feedforward neural network-random forest-recursive feature elimination algorithm, resulting in the development of two types of sensing arrays. Three 8-element sensing arrays were selected for detecting each cancer type. A 10-element sensing array was selected to differentiate among all three types of cancer simultaneously. The fabricated sensor arrays were exposed to simulated urine from cancer patients for 6 h at 45 °C. The urine of cancer patients was simulated through the addition of cancer-related VOCs to urine samples from five healthy individuals. The three 8-element arrays achieved AUC-ROC values of 1.00, 0.83, and 0.96 in the training set for bladder, colorectal, and PCa, respectively. Sensing elements exhibited a VOC detection threshold of 2.0 × 10^−4^ mg/cm^3^. The 10-element array achieved discrimination accuracy exceeding 85.0% after extracting RGB values in the training set. In the test set, an accuracy of >80.0% was achieved for bladder cancer and PCa, but only an accuracy of 58% for colorectal cancer was reached. Potential issues that undermined the sensor’s performance in the test set were the low number of healthy individuals that provided clinical urine samples for spiking and the limited diversity of the data the ML model was trained with. To facilitate POCT, the trained neural network model was incorporated into a smartphone application designed for RGB value extraction and results interpretation [116]. Another VOC fingerprinting approach was a biomimetic sensor array comprising 6 human olfactory receptor-embedded nanodiscs for PCa diagnosis. When a cancer-associated VOC binds to a receptor, the receptor undergoes a conformational change that alters the local environment of tryptophan residues and decreases tryptophan residue fluorescence intensity. Urine autofluorescence was normalized to increase the signal-to-noise ratio. Urinary VOC relevance was evaluated through correlation of olfactory receptor response with the Gleason score of cancers. Clinical urine samples from healthy individuals and PCa patients (*n* = 290) were used to train a machine learning model for prostate cancer diagnosis based on fluorescence intensity readouts. Classification performance was evaluated through conducting five-fold cross-validation using three different ML algorithms: logistic regression, random forest, and extreme gradient boosting. The Random Forest model achieved superior performance between all three models with an AUC-ROC of 0.964, an accuracy of 0.890, a precision of 0.928, a recall of 0.856, an F1-score of 0.889, a specificity of 0.927, and a positive trend with the Gleason score demonstrating closer association with the gold-standard histological tumor grade than PSA detection. However, it should be taken into consideration that when VOCs were initially evaluated for their viability as biomarkers, olfactory receptors were selected for correlating well with the Gleason score and that no apparent correlation could be established between olfactory receptor responses and serum PSA levels [117].

Moreover, a SERS-based immunoassay combined with deep learning for the monitoring of drug efficacy in HER2-overexpressing breast cancer was also developed. The principle involves Raman reporter-tagged Au@Ag SERS probes that were conjugated with antibodies to capture urinary EVs. SERS tag signals were analyzed using a deep neural network providing also determination of exosomal levels. The neural network was trained on 1000 randomly selected points from the spectra of each of the DTNB, 4-MBA, 2-MMTAA, and TFMBA SERS tag samples and 500 points from the spectrum of a 1:1:1:1 mixture of the SERS tag samples. The baseline of the silicon wafer’s spectrum was taken into consideration by randomly selecting 100 points and including them in the training set. Subsequently, the algorithm was validated in sets of SERS tag mixtures in varying ratios. The system was applied to urine samples from mice treated with trastuzumab and showed a strong correlation with traditional qRT-PCR and Western blot results [74]. Another report involves a label-free SERS method to discriminate Renal Cell Carcinoma (RCC) from healthy control samples using urine samples. The principle involves the use of 1 μL of urine droplets on dry solid silver nanoparticle substrates, focusing on vibrational bands associated with major components like urea and creatinine, followed by chemometrics and machine learning classification. SERS spectra were recorded with a 10-s acquisition time, while 50 spectra from different points were collected for each sample. In more detail, Principal Component Analysis combined with Linear Discriminant Analysis (PCA-LDA) and Support Vector Machine (SVM) (machine learning) were used for spectra analysis to discriminate between 50 male RCC patients, as validated through histopathological examination, and 44 male healthy donors, with PCA-LDA being more advantageous for this study. The PCA-LDA was validated through the leave-one-out cross-validation technique. Employing PCA-LDA, a discrimination accuracy of 100% and an 88% accuracy in discriminating between different RCC stages was achieved using seven principal components selected for showing the most significant differences between classes. The principal component selection step preceded cross-validation, instead of the standard nested cross-validation technique, thus introducing selection bias into the 88% figure. Alternatively, the SVM approach using a 94-segment cross-validation yielded a training accuracy of 100%, a validation accuracy of 99% for discriminating between RCC and healthy controls, and an 80% accuracy for discriminating between stages. As the SERS spectra of biological liquids strongly depends on molecules affinity for the plasmonic substrate, this method is a promising classification tool [118]. Moreover, a new diagnostic system utilized a 3D evolutionary gold nanoarchitecture (3D-EGN) for liquid-phase SERS profiling in urine for classifying cancer from normal groups. The principle involves forming a Au nanosponge on a 96-well plate followed by decoration of Au nanoparticles that enhanced light scattering. The platform was evaluated using malachite green showing an LOD of 1.23 nM. Machine learning algorithms based on Python and supervised convolution neural network (CNN) deep learning using Matlab was further applied for multiclass classification providing an accuracy of 95.6%. The CCN achieved sensitivities of 91.3, 95.6, 100, 100, and 91.3% and specificities of 98.9, 100, 98.9, 98.9, and 97.8% for normal, pancreatic, prostate, lung, and colorectal cancer urine samples, respectively. The corresponding AUC for normal samples was 0.99 and for each cancer type it was 1, 0.99, 0.99, and 0.98, respectively. The machine learning algorithm was trained on 80% of the total sample dataset and was tested in the remaining 20%. The samples were distributed randomly in sets. To account for imbalanced classes, the K-nearest neighbor oversampling with noise reduction (KNNOR) algorithm was used to oversample the minority class. Five-fold cross-validation was conducted in quintuplicate. The CNN training and test sets were produced in the same way as for the machine learning algorithm. To prevent overfitting, random undersampling was implemented. SERS spectra were recorded for 60 s and the system was applied to 218 clinical human urine samples (56 normal samples, 66 pancreatic, 39 prostate, 34 lung, and 23 colorectal cancer samples) [119]. In a similar approach, 218 urine samples from healthy individuals (*n* = 56) and patients with pancreatic (*n* = 66), prostate (*n* = 39), lung (*n* = 34), and colorectal (*n* = 23) cancer were analyzed through SERS metabolomic profiling and CNN-driven spectra analyses, achieving a diagnostic accuracy of >99%, sensitivity >98.6%, specificity >99.2% and an AUC of 0.99 for all cases. To address class imbalances, spectral data augmentation was implemented through simple physics-consistent perturbation of peaks in the spectra of minority classes within each training split. The detection was accomplished by using a highly sensitive plasmonic nanovine network structure (PNNS) placed onto a paper substrate and the conventional Raman reporter dye methylene blue, creating a urine strip-type diagnostic platform, while urine samples were analyzed without any pre-treatment. A volume of 10 μL of the sample was used after drying for a 2-s SERS measurement. Finally, a panel of urine metabolites was proposed for classification. At 633 nm the LOD was 0.83 nM, while at 785 nm an LOD of 0.74 nM was achieved [120]. Furthermore, Shoukat et al. 2026 combined electrochemical deposition-based-SERS with CNN analysis for the detection of multiplexed kidney disease biomarkers-proteins in urine. Electrochemical redox reactions were used for the deposition of plasmonic metals onto the sensor’s surface to fabricate SERS substrates. The SERS platform had a low LOD of 8.2 pg/mL for cytochrome C protein. Classification and regression CNN algorithms were trained using SERS spectra of the three proteins at different concentrations and 28 spectra of electrolyte solutions to account for the background of urine samples. Prior to training, the spectra were min-max normalized, and ANOVA was performed to detect measurements with significant differences between groups. As a result, 13 wavenumbers were used for feature selection from the training set. Five-fold cross-validation comparison using or omitting feature selection demonstrated that feature selection classification models were superior with respect to accuracy and stability. Occlusion sensitivity analysis determined that the selected spectral signatures were meaningful to the model’s predictions. The classification algorithm showed accuracy of 93.8% for albumin, 96.8% for transferrin, and 96.8% for IgG. CNN-based regression models for urinary protein quantification trained using a 70% train set and 30% test set, achieved R^2^ values of 0.9321 for albumin, 0.9848 for transferring, and 0.9957 for IgG. Spiking experiments in 5 artificial urine samples demonstrated recovery rates of 79.9–115.5%. The EC-SERS sensor performance was validated against ELISA using 15 clinical urine samples. The sensor achieved recovery rates from 75.0–125.9% for albumin, 74.2–124.5% for transferrin, and 81.5–115.0% for IgG [121]. Yin et al. 2025 manufactured a SERS-based array for the one-step isolation and digital counting of EVs. Single EVs (sEVs) were isolated by an EV-imprinted gold nanohole (EI-AuNH) array that selectively traps individual sEVs and spatially enhances their Raman spectra. A CNN model was then developed for digital counting of EVs for each patient based on the obtained spectra. The CNN model used a 70:30 training-test split on data that was first filtered using Smart-Filter to remove invalid spectral data (from incomplete or missing small extracellular vesicles) and then had the redundant observations (originating from the glass slide substrate or the APBA internal standard) removed. The total analysis time was about 40 min. This array demonstrated an accuracy of 97.37% in early diagnosis of bladder cancer in a cohort of 76 participants. The clinical cohort comprised 21 healthy volunteers and 55 bladder cancer patients [122].

#### 2.8.2. Electrochemical Sensors

Prithweeraj et al. 2026 combined electrochemical protein biosensing with machine learning-assisted classification for multiplexed breast cancer biomarker analyses. In this study, impedance and voltammetric data were collected from the targeted breast cancer-related proteins in urine, namely matrix metalloproteinase-9 (MMP-9), ALP, BCA-225 225/BRST and haptoglobin, and analyzed using SVM and neural networks. The SVM model achieved binary classification accuracies of 90.91% (MMP-9), 77.27% (ALP), 89.47% (BCA-225), and ~100% (Haptoglobin) in artificial urine samples. Multi-class classification for the SVM model provided 95.45%, 90.91%, 84.21%, and ~100% accuracy for MMP-9, ALP, BCA-225, and haptoglobin, respectively, while the neural network achieved multi-class classification accuracy of 46%, 75.73%, 96.97%, 76.04% for the same four biomarkers. The machine learning models were trained on 80% of the available data and tested using the remaining 20%. The SVM model optimized using hyperparameter tuning and the best results were selected through five-fold cross-validation. To improve the machine learning models’ performance in view of the limited dataset, measurements in triplicate for each biomarker concentration, an additional 100 data points were generated within the same range using interpolation techniques from scipy and the interpolate library [123].

Subsequently, electrochemical sensing systems have also been coupled with VOC profiling systems through multi-modal “electronic tongues” (eTongue) and “electronic noses” (eNose). A combination of an eNose and an eTongue was used for detection of PCa. The measurement involved the analysis of VOCs both from exhaled breath and urine samples using gas sensor arrays, while urine was simultaneously analyzed via CV on carbon and gold SPEs. Data fusion and machine learning (SVM, KNN, Random Forest, and Decision Trees) were used for classification, each paired with Discriminant Function Analysis (DFA) or PCA to reduce dimensionality. Each model’s performance was evaluated using a five-fold cross-validation. The data fusion of the data obtained using these sensors detected PCa with 97.3% accuracy, 100% sensitivity, and 97.8% specificity, while the authors reported that the combined sensors provided higher levels of discrimination than individual modalities [124]. Acevedo et al. 2024 also utilized a combined eNose and eTongue to evaluate urinary VOCs for PCa screening among patients. The integration of 15 gas-sensing channels and 8 electrochemical channels into the system provided a means for performing highly multiplexed biochemical analyses. A cohort of 113 cases comprised a PCa group (*n* = 66) and a control group (*n* = 47) which contained patients with prostate-related diseases and healthy individuals. The dataset was split into a 50% training set, 25% validation set, and a 25% test set. Different machine learning algorithms were implemented: Quadratic Discrimination Analysis, Naive Bayes, SVM, KNN, Random Forests, and Decision Trees. The algorithms were validated using five-fold cross-validation. The authors found that eNose provided 100% classification accuracy using the PCA-SVM model, whereas eTongue provided 92.9% accuracy using the DFA-k-nearest-neighbour model [125]. Yuskina et al. 2025 also developed an eTongue composed of 25 cross-sensitive sensors including PVC-plasticized membranes, metallic electrodes, and chalcogenide glass sensors for non-invasive screening of prostate and kidney cancers. Three datasets were tested: scaled signals, ratio signals, and a dataset from feature extraction accomplished through partial least squares regression–discriminant analysis. Machine-learning algorithms, including SVM, Random Forest, k-nearest neighbors, Logistic Regression, and extreme gradient boosting, were applied to process the multidimensional patterns of ionic responses and redox responses derived from the sensors’ measurements to provide classification accuracies of 77–87% across PCa (*n* = 39), kidney cancer (*n* = 38), and control groups (*n* = 39). The algorithms were validated on 1000 iterations of random 70:30 train–test dataset splits. The platform requires approximately 2 min per sample without extensive sample preparation, which highlights its utility for rapid clinical screening [126].

## 3. Discussion

Recent advancements in nanotechnology-enhanced (bio)sensors for new urinary cancer biomarkers are moving towards non-invasive point-of-care testing (POCT). Urine has emerged as an ideal candidate for liquid biopsy due to its high accessibility and rich composition of metabolites, proteins, nucleic acids, extracellular vesicles, and other molecules. However, the main challenge still remains the complex and variable matrix of urine and the extremely low concentration of target analytes.

To address the above limitations, the integration of novel nanomaterials, such as metal–organic frameworks (MOFs), nanozymes, and carbon-based nanostructures, has significantly improved analytical performance by enhancing the surface area for biorecognition and accelerating electron transfer improving electrochemical or optical signals. One significant trend also observed is the construction of enzyme-free sensors. While enzymes provide specificity, they are often vulnerable to matrix interferences and have high fabrication costs. The development of nanozymes, e.g., metal doped-MOFs or metal nanoparticles, and molecularly imprinted polymers (MIPs) has offered robust alternatives with high stability and anti-interference performance even in complex urine matrices. For instance, sarcosine (SAR) detection has reached sub-picomolar levels using self-powered wearable biofuel cells and dual-recognition binding mechanisms. Similarly, the use of dual-mode analysis (e.g., combining colorimetric and fluorometric signals, or SERS with fluorescence) improved the accuracy of the sensors by reducing false-positive results, which is critical for early cancer diagnosis.

Comparative evaluation of urinary metabolite and small-molecule biosensors and sensing systems for the detection of nucleic acids, proteins, extracellular vesicles, including exosomes, and other biomarkers in urine are presented in Table 1 and Tables S1–S6. To account for validation in different matrices (clinical urine, spiked urine, animal urine, and artificial urine samples) and provide a meaningful comparison, the figures reported on Table 1 and Tables S1–S6 correspond to measurements in buffer solution and reflect the sensors’ performance under optimal conditions. Only few reports include calibration graphs using artificial urine or real urine. The comparison of the recently developed biosensors for urinary metabolites—primarily focusing on the prostate cancer (PCa) biomarker sarcosine (SAR) and the gastric cancer biomarker 4-hydroxybenzoic acid (4-HBA) revealed the influence of nanotechnology in analytical performance, especially the detectability and the speed of the analysis. For sarcosine detection, traditional enzyme-based cascades utilizing sarcosine oxidase (SOx) show variable performance depending on the transducer and labels used. Colorimetric sensors achieved high LODs up to 1 μM, requiring 15–60 min of analysis time. The incorporation of metal–organic frameworks (MOFs) dramatically improved the detectability. For example, encapsulating SOx and horseradish peroxidase (HRP) within a zirconium MOF (Zr-MOF) with peroxidase-like activity lowered the LOD to 0.44 μM in colorimetric mode and 0.21 μM in fluorometric mode, though the analysis time was 51 min. Similarly, replacing HRP with bimetallic Fe/Co-MOF nanosheets, possessing also peroxidase-like activity, facilitated color development within 20 min, yielding a total analysis time of 30 min and an LOD of 0.324 μM. The introduction of fluorescent carbon dots (CDs) as dual-mode labels has further improved the detectability. A dual-mode sensor using Fe-doped CDs with peroxidase-like activity demonstrated an LOD of 0.17 μM (colorimetric) and a low LOD of 0.023 μM for the fluorometric mode with a 50-min analysis time. This represents a 10-fold improvement in fluorometric detectability compared to a similar dual-mode platform based on organic–inorganic hybrid nanoflowers, which yielded a fluorometric LOD of 0.226 μM in 45 min. The trend in POCT to lower the cost of the sensors is towards enzyme-free biosensors. A molecularly imprinted polymer (MIP) Zn/Ce-zeolitic imidazolate framework (Zn/Ce-ZIF) nanozyme sensor, an alternative to SOx, achieved a similar LOD of 1.32 μM within 60 min, while the integration of an enzyme-mimetic Cu-MOF with riboflavin lowered the LOD and the analysis time to 3.31 nM and 30 min, respectively. Combining electrochemical transduction on reduced graphene oxide nanoribbons (rGONRs) with an LOD of 0.04 nM within 30 min was accomplished, highlighting the enhanced detectability of the electrochemical sensors over other techniques such as colorimetry, which yielded a 2.0 μM LOD on the same platform. Similar, the use of MIP and CDs for the detection of 4-HBA provided superior analytical performance compared to other nanostructures. In addition, these novel peroxide-like nanomaterials have shown increased thermal and storage stability than free enzyme solutions and stability up to 20–30 days.

For urinary protein biomarkers, electrochemical, SERS-based sensors and optical fibers provided the lowest LODs. Enhanced sensitivity was attributed to the use of carbon nanotube/MOF, AuNPs, GO and MIPs. The enzyme-free sensors also showed greater stability in various conditions. However, they often require longer incubation times. In contrast, electrochemical sensors offered the fastest analysis time with enhanced simplicity. As for nucleic acids analysis, HRP-based signal amplification in combination with an electrochemical platform and specific DNA probes exhibited LODs at the attomolar level. Nucleic acid analysis presented includes amplification methods namely Recombinase Polymerase Amplification (RPA), Hybridization Chain Reaction (HCR), CRISPR-Cas collateral cleavage, Exponential Amplification Reaction (EXPAR), and digital droplet EXPAR (ddEXPAR). These methods and especially the DNA polymerase are strongly influenced by primer dimerization, non-specific hybridization, and urinary matrix inhibitors. Isothermal amplification techniques such as RPA which are performed at ambient temperature favors primer-primer annealing and non-specific bindings, resulting in high background noise and reduced target amplification efficiency. Platforms such as AVATAR mitigate this by immobilizing forward primers onto magnetic nanoparticles, physically separating primers in the liquid phase to prevent liquid-phase primer dimerization while maintaining single-copy detection limits. HCR is prone to spontaneous hybridization between hairpins and highly sensitive to secondary structures, leading to high background signal, while EXPAR is more susceptible to non-specific amplification than ddEXPAR. To prevent non-specific cross-hybridization, specialized reporter systems utilize γPNA (gamma peptide nucleic acid) hybrids. On the other hand, CRISPR-Cas method exhibits high specificity. However, in protein-targeting CRISPR platforms (e.g., Immuno-CRISPR for CXCL9/CXCL10), non-specific binding of antibody-DNA barcode conjugates to non-target matrix proteins can activate Cas12a trans-cleavage, causing false positives. Incorporating magnetic bead enrichment and extensive washing steps eliminate unbound barcode conjugates, reducing non-specific background and raising the signal-to-noise ratio. Finally, urine is not a friendly matrix for enzymes because it contains substances at high concentrations, such as urea and proteins; DNases; and RNases and presents fluctuations in ionic strength and pH that influence the activity of DNA/RNA polymerases, while high ionic strength strongly affects nucleic acid hybridization leading to lower efficiency of the above methods.

The choice of analytical matrix may alter biosensor performance due to varying background interference, ionic strength, pH, refractive index, and non-specific binding. Buffer solutions provide controlled ionic strength and pH without background organic interference, artificial urine lacks interreferences by macromolecules such as protein, lipids and metabolites, whereas real-urine samples are subjected to pretreatment like centrifugation and filtration that alter the final results [31,89,107,111,127]. Evaluating the LOD in buffer and in real urine environment, the biological matrix influences the analytical response in some cases, whereas for specific biomarkers there is no influence. Some examples include: (i) in a microRNA colorimetric assay, the LOD degraded from 0.43 nM in pure buffer to 0.62 in urine [65], (ii) in a self-powered wearable chip for sarcosine, the sensor achieved an ultralow LOD of 0.85 pM in buffer which rose to 0.15 μM in urine due to matrix interference [7], whereas (iii) an electrochemical sensor exhibited an ultralow LOD of 4 aM in both buffer and urine [107]. In most reports, however, LODs were determined in buffer and only recovery and repeatability studies were performed in real urine samples. Recovery samples were mostly conducted in diluted urine. Moreover, in several electrochemical and luminescent platforms, the sensor’s dynamic range is far below native urinary concentrations 10^−7^–10^−1^ ng/mL compared to the native range of 1–5 ng/mL. To analyze real samples, the authors mainly dilute urine 10^4–^10^8^ times. While this eliminates matrix interferences, it artificially shifts a native picomolar/nanomolar clinical sample into an attomolar measurement window after dilution. This represents diluted-sample handling rather than the direct detection of native attomolar level of biomarkers in raw biological fluids [56,85]. Assays that successfully achieve reproducible sub-femtomolar performance rely on magnetic capture or microfluidic enrichment from larger initial sample volumes to satisfy also Poisson sampling requirements [50,111]. Blank variations are increased in urine environment compared to buffer solutions and must be taken into consideration for LOD determination. As an example, EC/ECL and SERS platforms exhibited differences in the slope of calibration graphs and baseline variations when a biological fluid was used [7,107].

A challenge faced by SERS-based techniques is low signal-to-noise ratios negatively affecting analytical performance. To this end, various noise management techniques have been developed such as Wiener estimation spectral reconstruction, modifying probes using 2D-materials, and rough-cutting the end-face of fibers in optical fiber SERS systems, although application in urine liquid biopsy has not yet been reported. The Wiener estimation may be used to synthesize narrow-band measurements from low signal-to-noise ratio spectra to eliminate noise [128]. A source of noise for SERS spectra is signal non-uniformity across substrates. An AuNP/graphene substrate yielded a 279% increase in signal-to-noise ratio. 2D hybrid substrates also remove background fluorescence via quenching or energy transfer [129]. Another source of noise is the background signal generated by the optical fiber material. It is reported that rough cutting the end-face of the fiber material reduced noise by approximately 32% compared to a flat surface and simultaneously increased light collection efficiency from 1.2% to 8.5%, resulting in a 27-fold increase in the signal-to-noise ratio [130].

Another important trend is the integration of Machine/Deep Learning (ML/DP) and Artificial Intelligence (AI), which has revolutionized the data interpretation of the sensors output, by processing of complex data from single and multisensory platforms, e.g., data from SERS spectra. These algorithms facilitated the extraction of unique biochemical “signatures” or fingerprints from urinary components such as metabolites, volatile organic compounds (VOCs) and protein patterns, achieving diagnostic accuracies often exceeding 90%. The most widely used model was Support Vector Machine, paired with flow cytometry, SERS and multiple electrochemical sensors, including e-Nose and e-Tongue approaches. Convolutional Neural Networks were particularly useful in interpreting spectral data or complex image data, as well as digital counting of single EVs. Other algorithms that appeared, mainly in studies comparing the performance of multiple algorithms, were Random Forest, XGBoost, Logistic Regression, Naive Bayes, Quadratic Discriminant Analysis, k-Nearest Neighbors, Decision Trees and Discriminant Function Analysis. Several works incorporated data processing techniques, prior to implementing ML-based data interpretation with statistical tools (ANOVA, PCA), mainly for dimensionality reduction, ML statistical/classification methods for feature extraction (discriminant function analysis, partial least squares regression-discriminant analysis), and attempted to improve on generalizability despite the limited size of clinical cohorts and imbalanced classes by generating synthetic data (scipy’s interpolate library), balancing classes (KNNOR, physics-consistent spectral perturbation), and random undersampling to prevent overfitting. Additional data-processing steps were min-max normalization and baseline correction to remove background signals. The standard validation approach for ML models was five-fold cross-validation, splitting the training dataset into 5 sub-sets, training on 4 sets, and performing validation on the remaining set. The most common train/test splits were 70%/30% and 80%/20%. Despite these measures, several works reported a significant decrease in ML model performance when transitioning from the training set to the test set, hinting at limited generalizability. Furthermore, the miniaturization of these platforms into smartphone-assisted devices provided the potential for decentralized clinical applications.

However, the interpretation of urinary biomarkers can also be affected by non-malignant conditions and patient-related factors. Urinary tract infection and inflammation, benign urological disease, hematuria and impaired renal function can alter urinary composition [6], while diet, hydration and medications may also influence the levels of urinary components [131]. These factors should therefore be considered when evaluating the cancer specificity of individual biomarkers or biomarker profiles. In addition, the performance, the accuracy and reproducibility of sensing platforms for urinary biomarker analysis can be significantly influenced by pre-analytical factors related to urine sample handling, including the time of collection, hydration, pH, specific gravity, centrifugation, filtration, storage, freeze–thaw cycles, and normalization techniques [131,132]. Since many of the reviewed platforms were developed primarily as analytical proof-of-concept systems, the absence of relevant disease-control groups should be considered when interpreting claims of clinical specificity or screening performance.

Urinary assays evaluated in animal models should be interpreted as preclinical proof-of-concept rather than direct evidence of human diagnostic performance. Mouse cancer models differ in their ability to reproduce human tumor development, immune responses, and the tumor microenvironment, depending on the experimental model used. Therefore, urinary biomarkers and analytical performance identified in animal models require confirmation in human samples before their clinical diagnostic relevance can be established [133].

Despite the impressive analytical performance of the reported biosensors, the translation of novel biosensing technology into clinical routine practice remains limited by still the lack of comprehensive clinical validation studies. At a minimum, evaluation in clinically characterized human urine samples, including appropriate disease-control groups, comparison with an appropriate reference method, assessment of diagnostic performance, and demonstration of reproducibility in the urine matrix, should be required before a sensing platform can be considered a candidate for clinical screening or point-of-care testing.

## 4. Conclusions

In conclusion, the development of nanotechnology-based biosensors and the use of novel urine biomarkers have provided highly sensitive tools for the early diagnosis and monitoring of various cancers through urinary analysis. The application of plasmonic nanostructures, electrochemical transducers, and synthetic biorecognition elements with outstanding properties, either optical or electrochemical with high surface areas, has pushed detection limits to the femtomolar and attomolar levels, particularly for nucleic acids and exosomal biomarkers. These advancements offer a promising alternative to invasive biopsies and costly imaging techniques.

Looking forward, the future of urinary cancer sensing lies in the standardization of sample-processing workflows, validation of the sensing methods and the construction of robust multiplexed analytical devices. While high-performance sensors for PSA, SAR, and miRNAs have been successfully demonstrated in laboratory settings, the path to clinical routine involves bridging the gap between innovative prototypes and large-scale clinical technology validation. By combining the high specificity of molecular biology with the robustness of nanotechnology and the interpretive power of AI, next-generation sensors are promising to become the state of the art in personalized medicine and global health screening.

## Figures and Tables

**Table 1:** Comparative table of all sensing principles for the detection of various urine biomarkers.

Technique	Biomarker	Analytical Performance/Limit of Detection (LOD) & Analysis Time
Optical/Colorimetric	Sarcosine	LODs: 1.14 pM–10 μM Lowest LODs: 1.14 pM (in buffer, R^2^ = 0.9928, RSD < 5%) & 10.0 nM (in buffer, R^2^ = 0.9959, RSD < 4.6%, Recovery 98.7–101.5% with RSD < 2.03% in urine) [7,29] Analysis time: 15–60 min
Optical/Fluorescence	Sarcosine	LODs: 3.31 nM–0.226 μM Lowest LOD: 3.31 nM (in buffer, R^2^ = 0.992, RSD:1.63%, Recovery 96.7–106.1% with RSD < 5% in urine) [34] Analysis time: 20–50 min
Optical/Chemiluminescence	Sarcosine	LOD: 230 μM (in buffer, R^2^ = 0.992, RSD < 5%, Recovery 92.3–115.9% with RSD < 10% in urine) [30]; analysis time: 23 min
Electrochemical	Sarcosine	LODs: 166 fM–0.35 μM Lowest LOD: 166 fM (in buffer, R^2^ = 0.9971, RSD: 2.5%, Recovery 97.0–106.7% with RSD < 3.8% in urine) [18] Analysis time: 20–50 min
Optical/Fluorescence	Citrate	LOD: 3.5 nM (in buffer, R^2^ = 0.9802, Recovery 99.3–100.1% with RSD:1% in urine) [42] Analysis time: 5 min
Optical/Luminescence	Vanillylmandelic acid	LOD: 340 pM (0.41 nM in artificial urine & 0.34 nM in urine, R^2^ = 0.9760 and 0.9806, Recovery 99.6–106.9% with RSD < 7.8% and 100.1–103.5% with RSD < 8.8 in artificial urine & urine) [46] Analysis time: 10 s
Optical/SERS	Urinary Metabolites	LOD: 740 pM (in buffer, R^2^ = 0.99, RSD < 11%) [120] Analysis time: 2 s
Optical/Fluorescence	PSA	LOD: 14.5 pg/mL (in buffer, R^2^ = 0.99, Recovery 95.7–104.0% with RSD < 1.7% in urine) [21] & 27.7 pg/mL [66] Analysis time: 4–135 min
Optical/Dual Optical Microfibers	PSA	LOD PSA: 3.97 fM (in buffer, R^2^ = 0.994, RSD < 4.4%) [80]
Electrochemical	PSA	LODs: 0.10 fg/mL (in buffer, R^2^ = 0.998, RSD < 4.4%, Recovery 97–107% in artificial urine) [94] & 0.35 pg/mL (in buffer, R^2^ = 0.9916) [93]
Optical/SPR	Nuclear matrix protein 22 (NMP22)	LOD: 0.092 ng/mL (in buffer, R^2^ = 0.992, Recovery 100.3–108.3% in urine) [79]
Electrochemical	NMP22 protein	LODs: 2.96 × 10^−2^ fg/mL–3.3 fg/mL Lowest LOD: 2.96 × 10^−2^ fg/mL (in buffer, R^2^ = 0.9995, RSD < 3.7%, Recovery 80.1–103.8% with RSD < 4.7% in urine) [85]
Optical/SERS	Hyaluronidase	LOD: 3 × 10^−4^ U/mL (in buffer, R^2^ = 0.9991) [77]
Electrochemical	Hyaluronidase	LOD: 0.414 U/mL (in buffer, R^2^ = 0.992) [113]
Optical/Colorimetric	microRNAs	LOD: 0.62 nM in urine & 0.43 nM in buffer (in urine, R^2^ = 0.9884, Recovery 75.8–100) [65] Analysis time: 65 min
Optical/Fluorescence	microRNAs	LODs: 3 fM (in buffer, R^2^ = 0.937) [67] & 17.3 fM [66] Analysis time: 50–135 min
Optical/SERS	microRNAs	LOD: 0.5 nM in buffer [20]
Optical/SPR	microRNAs	LOD: 0.36 pM (in buffer, R^2^ = 0.9923) [78]
Electrochemical	microRNAs	LODs: 4 aM–100 fM Lowest LODs: 4 aM in both buffer (R^2^ = 0.96) and urine (R^2^ = 0.97) [107] & 0.12 fM (in buffer, R^2^ = 0.9877, RSD:1.05%) [106]
Electrochemical	PCA3 RNA	LOD: 1 fM in buffer (R^2^ = 0.91) and 20 fM (R^2^ = 0.95) in artificial urine [103]
Optical/Dual Optical Microfibers	PCA3 RNA	LOD: 15.6 fM (in buffer, R^2^ = 0.995) [80]
Optical/Fluorescence	EVs	LODs: 10^3^ (EVs isolated from cell culture) [15]—2.95 × 10^3^/4.23 × 10^4^ EVs/mL (EVs isolated from cell culture, R^2^ = 0.782–0.905) [68] Analysis time: 60 min
Optical/Colorimetric	EVs	LOD: 8.5 × 10^8^ EVs/mL (EVs isolated from cell culture, R^2^ = 0.9942) [72] Analysis time: 2 h
Electrochemical	EVs	LODs: 29 EVs/μL (EVs isolated from cell culture, R^2^ = 0.9962, Recovery 99–103% with RSD < 2.13% in artificial urine) [110] & 483 EVs/µL (EVs isolated from cell culture, RSD = 4.3%) [109] Analysis time: 10–30 min

## Data Availability

The original contributions presented in this study are included in the article/Supplementary Materials. Further inquiries can be directed to the corresponding authors.

## Supplementary Materials

The following supporting information can be downloaded at https://www.mdpi.com/article/10.3390/s26196258/s1, Table S1: Sensors and sensing systems for the detection of metabolites and small molecules in urine; Table S2: Sensors and sensing systems for the detection of proteins and antigens; Table S3: Sensors and sensing systems for the detection of nucleic acids: circulating tumor DNA (ctDNA), RNAs & microRNAs; Table S4: Sensors and sensing systems for the detection of extracellular vehicles (EVs); Table S5: Sensors and sensing systems for the detection of volatile organic compounds (VOCs); Table S6: Sensors and sensing systems for the detection of RNA nucleosides & cancer cells.

## Abbreviations

The following abbreviations are used in this manuscript:

AUC	Area under the curve
AuNPs	Gold nanoparticles
CDs	Carbon dots
CEA	Carcinoembryonic antigen
ctDNA	Circulating tumor DNA
CV	Cyclic voltammetry
CVs	Coefficients of variation
CXCL9	Chemokine ligand 9
CXCL10	Chemokine ligand 10
DFA	Discriminant function analysis
DPV	Differential pulse voltammetry
dsDNA	Double-stranded DNA
EIS	Electrochemical impedance spectroscopy
ELISA	Enzyme-linked immunosorbent assay
EVs	Extracellular vesicles
FITC	Fluorescein isothiocyanate
FRET	Förster resonance energy transfer
GCE	Glassy carbon electrode
GO	Graphene oxide
HRP	Horseradish peroxidase
HPLC	High-performance liquid chromatography
LFA	Lateral flow assay
LC	Liquid chromatography
LDA	Linear discriminant analysis
MIP	Molecularly imprinted polymer
miRNA	microRNA
MOF	Metal–organic framework
MS	Mass spectrometry
NCs	Nanoclusters
NMP22	Nuclear matrix protein
PCa	Prostate cancer
PCA	Principal component analysis
PCA3	Prostate Cancer Antigen 3
PEC	Photoelectrochemical
PEG	Poly(ethylene glycol)
POCT	Point-of-care testing
PSA	Prostate-specific antigen
PSMA	Prostate-specific membrane antigen
Rf	Riboflavin
rGO	Reduced graphene oxide
ROC	Receiver operating characteristic
RPA	Recombinase polymerase amplification
RSD	Relative standard deviation
SAR	Sarcosine
SERS	Surface-enhanced Raman scattering
SOx	Sarcosine oxidase
SPCE	Screen-printed carbon electrode
SPR	Surface plasmon resonance
ssDNA	Single-stranded DNA
SVM	Support vector machine
SWV	Square wave voltammetry
TMB	3,3′,5,5′-tetramethylbenzidine
VOC	Volatile organic compound
VMA	Vanillylmandelic acid
ZIF	Zeolitic imidazolate framework
