# Supplementary material for: Advances in Urinary Liquid Biopsy: A Review of Emerging Sensing Platforms for Early Cancer Screening and Diagnosis
Source: Sensors (Basel). 2026 Oct 2;26(19):6258. doi: 10.3390/s26196258 (PMC13667545; doi:10.3390/s26196258)
Supplement: Supplementary file 1 [file sensors-26-06258-s001.zip › sensors-4549933-supplementary.pdf]

Supplementary Material

Advances in Urinary Liquid Biopsy: A Review of Emerging Sensing Platforms for Early Cancer Screening and Diagnosis

Eleni Lamprou*, Dimitrios Papakyriakou and Despina P. Kalogianni*

[1] Analytical Chemistry & Nanotechnology Group, Department of Chemistry, University of Patras, GR 26504, Patras, Greece[2]

* Correspondence: E.M.: eleni_lamprou@upatras.gr; D.P.K. kalogian@upatras.gr

Table S1. Sensors and sensing systems for the detection of metabolites and small molecules in urine.

REF.	TECHNIQUE	METHOD	BIOMARKER(S)	ANALYTICAL PERFORMANCE
OPTICAL: COLORIMETRIC & FLUORESCENT				
**10**	Optical: Fluorescence	ZIF/CDs@MIPs	4-Hydroxybenzoic acid	**LOD: 0.68 μM**; **Range**: 0-100 μM; **Recovery**: 97.7–106.2%; **RSD**: 0.80–2.95%; **IF**: 4.35; analysis time: 13 min
**17**	Optical: Dual-mode/ Fluorescence & Colorimetric	SOx/peroxidase-like Fe-CDs/DPD cascade	Sarcosine	**LOD: 0.023 μM (fluorescence) / 0.17 μM (colorimetric); Range:** 0.05– 120 μM; **Recovery**: 91.0%–109.2%; **RSD**: <4.9%; analysis time: 50 min
**22**	Optical: Colorimetric/ Smartphone	SOx/peroxidase-like Fe-MOF/TMB cascade for Au nanorod etching	Sarcosine	**LOD: 0.12 μM**; **Range**: 1–60×10^{-6} M; **Recovery**: 95.5–105.8%; **RSD**: 2.4–5.8%; analysis time: 15 min
**26**	Optical: Colorimetric/ Smartphone/ Paper-based	Oxidase/HRP/TMB cascade	Sarcosine	**LOD: 1 μM**; **Range:** 2–500 μM; **Recovery**: 95.4–108.7% and **RSD**: <3.5%; analysis time: 34–44 min
**27**	Optical: Dual mode/ Fluorescence & Colorimetric	SOx/HRP/Zr-MOF/PPD cascade and CD quenching	Sarcosine	**LOD: 0.21 μM (fluorescence) & 0.44 μM (colorimetric)**; **Range**: 0.5–200 μM **Recovery**: 97.3–104.2%; **RSD**: 0.78–3.75%; analysis time: 51 min
**28**	Optical: Colorimetric	SOx/peroxidase-like 2D Fe/Co-MOF]/TMB cascade	Sarcosine	**LOD: 0.324 μM**; **Range**: 1–400 μM; **Recovery**: 97.3–105.8%; **RSD**: 0.4–1.6%; analysis time: 30 min
**29**	Optical: Colorimetric/ Paper-based	SOx/peroxidase-like CDs/TMB cascade for distance-based detection	Sarcosine	**LOD: 10.0 nM***; **Range**: 12.5-35.0 nM; **Recovery:** 98.7-101.3%; **RSD**: <2.03%; analysis time: 30 min
**30**	Optical: Chemiluminescence	SOx/peroxidase-like Ag/Fe metal-organic gel/SOx/luminol cascade	Sarcosine	**LOD: 0.23 mM**; **Range**: 1-50 mM; **Recovery**: 92.3–115.9%; **RSD**: <10%; analysis time: 23 min
**31**	Optical: Dual mode/ Fluorescence & Colorimetric/ Swab test	SOx/peroxidase-like fluorescent $Cu_3(PO_4)_2$:Ce nanoflowers	Sarcosine	**LOD: 0.226 μM (fluorescence) & 0.120 μM (colorimetric)**; **Range:** 0.5–60 μM (fluorescence) & 0.18–60 μM (colorimetric); **Recovery**: 93.4–107.30%; **RSD**: 2.9–9.4%; analysis time: 45 min
**32**	Dual mode/ Colorimetric & Photothermal	SOx/CuS@CeO_2/TMB cascade with hot-carrier heat dissipation	Sarcosine	**LOD: 0.52 μM (colorimetric) & 0.57 μM (photothermal)**; **Range:** 1–100 μM; **AUC:** 0.990; **Sensitivity** 96.2%; **Specificity:** 96.4%
**33**	Optical: Colorimetric/ Smartphone	Zn/Ce-ZIF nanozyme with a molecularly imprinted shell and peroxidase-like core	Sarcosine	**LOD: 1.32 μM**; **Range**: 2–500 μM; **Recovery**: 95.2–106.8%; **RSD:** 1.9–2.5%; analysis time: 60 min
**34**	Optical: Fluorescence	Multilayered sandwich sensor peroxidase likeCu-MOF/Rf@BDC	Sarcosine	**LOD: 3.31 nM**; **Range**: 0.01–60 μM; **Recovery**: 96.71–106.07%; **RSD**: <5.0%; analysis time: 30 min

**35**	Dual mode/ Electrochemical & Colorimetric/ Smartphone	Rf@rGONRs/peroxidase-like TCPP(Fe)-Zn MOF/OPD	Sarcosine	**LOD: 0.04 nM (electrochemical) & 2.0 μM (colorimetric)**; **Range**: 0.001–500.0 μM (electrochemical) & 10–1000.0 μM (colorimetry); **Recovery**: 97.1–110.7%; **RSD**: 5.3% (electrochemical) & 4.2% (colorimetric)
**36**	Optical: Colorimetric/ Smartphone	Cellulose nanofiber/polyacrylic acid hydrogel with SOx/HRP/TMB cascade	Sarcosine	**LOD: 10 μM**; **Range**: 0–100 μM; analysis time: 20–30 min
**37**	Optical: Fluorescence	Fe(III)-quenched L-cysteine capped bimetallic copper-silver nanoclusters	L-Leucine	**LOD: 0.58 μM**; **Range**: 110–657 μM; **Recovery**: 84.9–100.6%; validated in saliva and urine
**38**	Optical: Fluorescence	Fe(III)-quenched L-cysteine capped copper nanoclusters	Valine	**LOD: 3.0 μM**; **Range:** 73–366 μM; **Recovery**: 94.85–101.16%
**40**	Optical: Dual mode/ Colorimetric & Fluorescence	Xanthyllium-bsead probe	Cysteine	**LOD: 31.8 μM (colorimetric) & 3.1 μM (fluorescence); Range:** 0–300 μM; **Recovery:** 90.7–106.3% (colorimetric) & 97.3–99.9% (fluorescence); **RSDs:** 7.92–10.11% (colorimetric) & 0.49–1.16% (fluorescence)
**42**	Optical: Fluorescence	N-doped CDs	Citrate	**LOD: 3.5 nM**; **Recovery**: 99.3–100.07%; **RSD**: 0.91–1.02%; analysis time: 5 min
**43**	Optical: Fluorescence	N,S-CDs@SiO2@MIPs for dynamic quenching and electron transfer turn-off sensing	4-Hydroxybenzoic acid	**LOD: 1.27 μM**; **Range**: 0–100 μM; **Recovery**: 97.87–113.6%; **RSD**: <2.9%; **IF**: 3.32; analysis time 18 min
**44**	Optical: Fluorescence	magnetic hollow Fe_3O_4/CDs@MIPs	4-Hydroxybenzoic acid	**LOD: 0.37 μM**; **Range**: 0–100 μM; **Recovery**: 95.0–106.7%; **RSD**: 0.8–2.8%; analysis time: 11.5 min; **IF**: 40.78
**46**	Optical: Dual/ Luminescence & Colorimetric/ Test-strip	14-metal Tb(III)nanoclusters for antenna effect-based detection	Vanillylmandelic acid	**LOD: 0.34 nM**; **Recovery:** 98.7–100.03%; **RSD:** 3.3–8.5%; analysis time: 10 s
**48**	Optical: Fluorescence	19-metal Cd(II)-Eu(III) nanoclusters for antenna-effect-based sensing	Biopterin	**LOD: 0.22 nM; Range:** 0-100 μM; **Recovery:** 94.05–113.73%; **RSDs:** 0.34–1.43%
OPTICAL: SURFACE-ENHANCED RAMAN SPECTROSCOPY (SERS)				
**16**	Optical: SERS & Fluorescence	3D DNA hydrogel disassembly	EpCAM	**LOD: 0.17 pg/mL (SERS) / 0.35 pg/mL (FL)**; **Recovery**: 85.8–98.3%; **RSD**: 2.4–5.7 %
**75**	Optical: SERS	PCA-PLS-DA / Au/ZnO nanorods	EpCAM, lipids, Amide III	**Accuracy: >99.6%**; analysis time: 30 min; sample volume: 5 μL
**118**	Optical: SERS	PCA-LDA / SVM (AgNP)	Urine metabolites	**Validation accuracy:** 99%; 50 spectra from different points were collected for each sample; analysis time: 10 s
**119**	Optical: SERS / machalite green	3D- gold nanoarchitecture / Deep Learning (CNN)	Metabolite profiling	**LOD: 1.23 nM**; **Accuracy:** 95.6%; analysis time 60 s

**120**	Optical: SERS	SERS (PNNS) with Deep Learning	23 Urinary Metabolites	**LOD: 0.74 nM**; **Accuracy: >99%**; analysis time: 2 s; 4 types of cancer
OTHER OPTICAL SENSORS				
**82**	Liquid crystal-filled transmission electron microscopy	dsDNA probe / Orientational transition	Platelet-derived growth factor (PDGF-BB)	**LOD: 2.63 pg/mL**; **Range**: 5–200 pg/mL; analysis time: 60 min
**83**	Optical: tapered optical fiber	AuNP-decorated tapered optical fiber	Human chorionic gonadotropin (hCG) hormone	**LOD: 0.03 mIU/mL** (urine); **LOQ**: 0.11 mIU/mL; **Range**: 1–180 mIU/mL
ELECTROCHEMICAL				
**7**	Dual mode/ Electrochemical & Optical	Self-powered Wearable Chip	Sarcosine	**LOD: 0.85 pM (EC) / 1.14 pM (colorimetric)**; **Range**: 10 pM–500 μM; RSD: <5%; smartphone
**8**	Electrochemical	MOF-derived graphitic carbon-decorated $NiVO_4$ modified GCE	Sarcosine	**LOD: 0.03 μM**; **LOQ**: 0.1 μM; **Recovery**: 95–105%
**18**	Electrochemical:EIS	Melamine-based COF/MIP modified electrode/ AuNPs/GCE	Sarcosine	**LOD: 0.166 pM**; **Range**: 500 fM–350 pM
**87**	Electrochemical: Chronoamperometry	Dual-mode nCuCd-MOF bioelectrode	Sarcosine, Creatinine	**LOD: 0.35 μM (sarcosine) & 0.20 μM (creatinine); Range:** 1–14 μM (sarcosine) & 5–105 μM (creatinine); **RSDs:** <5%
**88**	Electrochemical: piezoelectric	Molecularly imprinted hydrogel swelling and piezoelectric strain gauge transducer	Sarcosine	**Range:** 10^{-3}-10^{6} μM
**19**	Electrochemical: DPV	3D molecularly imprinted polymer (MIP) nanofilm integrated with an EGFET	Urinary Spermine	**LOD: 1.23 ng/mL**; **Range**: 0.1–1000 ng/mL; IF: 4.1; correlation with LC-MS: 0.98
**84**	Electrochemical	Laccase-mimicking Pt/Mn & MnO_2 nanozyme	5-hydroxy-indoleacetic acid (5-HIAA)	**LOD: 0.16 & 1.4 μM**; **Range**: 0.3–15 μM & 2–50 μM; **Recovery**: 94.1%; **RSD**: <5%
**86**	Electrochemical: Paper-based	Aptamers/ silver nanowires/electrodes/ CV	Epidermal Growth Factor Receptor (EGFR)	**LOD: 0.01 ng/mL**; **Range**: 0.01 ng/mL–1 μg/mL; **Recovery**: 94.8–97.4%; analysis time: 30 s
**89**	Electrochemical: EIS	CA/r-GO modified SPCE	Urinary Zinc (UZn)	**LOD: 7.33 ng/mL**; **Range**: 0.1–1000 ng/mL; 0.991 correlation with ICP-MS
**92**	Electrochemical: DPV	Nickel monoligand-MOF modified carbon paste electrode	Homovanillic acid (HVA)	**LOD: 0.08 μM**; **Range**: 0.1–70.0 μM; **Recovery**: 93.2–97.6%; **RSD** <2%
**101**	Electrochemical	MoS_2/gC_3N_4/CDs nanohybrids immunosensor	Human chorionic gonadotropin (hCG) hormone	**LOD: 0.015 ng/mL; Range**: 10^{-4}–80 ng/mL; **Recovery**: 99.5%; analysis time: 3 min

* Lowest LODs are highlighted in red

Table S2. Sensors and sensing systems for the detection of proteins and antigens.

REF.	TECHNIQUE	METHOD	BIOMARKER(S)	ANALYTICAL PERFORMANCE
OPTICAL: COLORIMETRIC & FLUORESCENT				
**21**	Optical: Fluorescence	Fluorescent caboxy-porous organic polymer-based immunoassay	PSA	**LOD: 14.5 pg/mL**; **Range** of 0–36 ng/mL; **Recovery**: 95.7–104.0%; **RSD**: 0.16–1.7%; analysis time: 4 min
**49**	Optical: Fluorescence	Turn-on sensing immunoassay based on CuNCs quenched by Graphene Oxide	Glial fibrillary acidic protein	**LOD: 32.0 pg/mL**; **Range**: 0.0–46.3 ng/mL; **Recovery**: 84.8–100.4%
**50**	Optical: Fluorescence & Visual LFA	Signal amplification via CRISPR Cas12a trans-cleavage of FRET-based reporters and LFA-AuNPs	CXCL9, CXCL10	**LOD: 0.6 pg/mL (CXCL9, fluorescence), 2.4 pg/mL (CXCL10, fluosecence) & 18 pg/mL (CXCL9, LFA), 39 pg/mL (CXCL10, LFA)**; analysis time: 90–93 min
**51**	Optical: Colorimetric & Visual LFA	Combined LFA-AuNPs (uPSA) and colorimetric dithizone-saturated membrane sensing	PSA and Zn	**Threshold value:** 2800 ng/mL (PSA) & 1400 ng/mL (Zn); **AUC**: 0.769; visual detection
OPTICAL: SURFACE-ENHANCED RAMAN SPECTROSCOPY (SERS)				
**77**	Optical: SERS	SERS (hyaluronic acid-AgNPs)	Hyaluronidase (HAase)	**LOD: 3×10^{-4} U/mL**; **Range:** 3×10^{-4}–3×10^{1} U/mL; **Accuracy:** 95.1%; **Specificity**: 95%; **RSD**: <5.47%
**121**	Electrochemical / Optical (SERS)	EC-SERS (AuNDs) / CNN	Albumin, transferrin, IgG	**LOD: 8.2 pg/mL**; Accuracy: 93.8-96.8%
OPTICAL: SURFACE PLASMON RESONANCE (SPR)				
**79**	Optical: SPR	Au-coated optical fiber / immobilized NMP22 antibody / RI-red shift	Nuclear matrix protein 22 (NMP22) (Bladder Cancer)	**LOD: 0.092 ng/mL**; **Range**: 0–25 ng/mL; **AUC**: 0.8961; **Specificity**: 76.7%; **Recovery**: 100.3–108.4%
OTHER OPTICAL SENSORS				
**80**	Optical: dual optical microfibers	LSPR (GNR / Au bipyramid)	PSA protein / PCA3 RNA	**LOD PSA:** 3.97×10^{-15} M; **LOD PCA3:** 1.56×10^{-14} M
ELECTROCHEMICAL				
**9**	Electrochemical	ZIF-8@MWCNTs@Chit@Fc@AuNPs	Nuclear matrix protein 22 (NMP22)	**LOD: 3.33 fg/mL**; **Range**: 0.01 pg/mL–^{10}00 ng/mL; **RSD**: 2.23%
**52**	Electrochemical: ECL	Indicator-free detection via conductivity-regulated bipolar electrochemiluminescence	Hyaluronidase (HAase)	**LOD: 0.13 U/mL**; **Range**: 1–60 U/mL; analysis time; 90 min
**85**	Electrochemical	Ordered Mesoporous Carbon label-free immunosensor/AuNPs/ carboxylate graphene-supported thionine nanocomposites	NMP22 protein (Bladder Cancer)	**LOD: 2.96×10^{-8} ng/mL**; **Range**: 10^{-7}–10^{-1} ng/mL; **Recovery**: 80.1–103.8%; **RSD**: 0.1–4.7%
**93**	Electrochemical: Photoelectrochemical immunosensor	MoS_2/N,S-CQDs/Bi_2S_3 nanoheterojunction	PSA	**LOD: 0.35 pg/mL**; **Range**: 0.001–50 ng/mL; **Recovery**: 97.4–103.0%; **RSD**: <1.54%

		deposited on fluorine-doped tin oxide electrodes		
**94**	Electrochemical	rGO-modified SPE	PSA	**LOD: 0.10 fg/mL**; **LOQ**: 1.04 fg/mL; **Recovery**: 97–107%; **RSD**: 4.02–4.57%
**96**	Electrochemical	Molecularly Imprinted Polymer (MIP)	NMP22 protein (Bladder Cancer)	**LOD: 0.077 pg/mL**; **Range**: 0.1 pg/mL–1 μg/mL; **Recovery**: 96.9–100.9%; analysis time: 20 min
**97**	Electrochemical: Amperometric	Cascade enzyme immunoassay microfluidic chip	PSA, Engrailed-2	**LOD: 0.154 pg/mL (PSA) & 0.146 pg/mL (Engrailed-2); Range:** 0.001–100 ng/mL; **Recovery:** 97.3–106.5%; **RSDs**: <9.01%
**98**	Electrochemical	Multiplexed 3D-rGO/AuNP	PSMA protein	**LOD: 5 pg/mL**; **Range**: 15–2000 pg/mL; **RSD**: ≤5%; analysis time: >30 min
**99**	Electrochemical	Fe_3O_4/APTES/GO Immunosensor	Engrailed-2 (EN2)	**LOD: 41 nM**; **Range**:75–150 nM; **AUC**: 0.65
**100**	Electrochemical: EIS	Label-free EIS (Anti-CEA/AuNPs)	CEA (Gastrointestinal)	**LOD: 4.4 pg/mL; Range**: 13.2 to 1×10^5 pg/mL; **Recovery**: 86–94%; real-time
**3**	Electrochemical	Ag@ZIF-90 GCE immunosensor	CA 19-9 (Pancreas)	**LOD: 9×10^{-5} U/mL; Range**: 10^{-4}–2000 U/mL; **Recovery**: >97.2%; **RSD**: <4.6%
**102**	Electrochemical: SWV	Tandem peptide/PNA probe/ DNA-walker biosensor	Cathepsin B (CB)	**LOD: 17.5 pg/mL**; Analysis time: 2–3 h
**113**	Electrochemical	Hyaluronic acid-degradable hydrogel	Hyaluronidase (HAase)	**LOD: 0.414 U/mL**; **Range**: with 2–25 U/mL
**123**	Electrochemical	SVM with Machine Learning	4 Protein Panel (Urine)	**Accuracy:** 84.2-100%

Table S3. Sensors and sensing systems for the detection of nucleic acids: circulating tumor DNA (ctDNA), RNAs & microRNAs.

REF.	TECHNIQUE	METHOD	BIOMARKER(S)	ANALYTICAL PERFORMANCE
OPTICAL: COLORIMETRIC & FLUORESCENT				
ctDNA				
**63**	Optical: Fluorescence	DNA/γPNA hybrid nanoreporter and FRET-based detection	ctDNA mutation/KRAS(G12D)	**LOD: 5.4 pM**; **Range:** 30–1000 pM analysis time: 5.75 h
RNAs & microRNAs				
**64**	Optical: Visual LFA	PDMS device for isothermal amplification and LFA-AuNPs	PSA & PCA3 mRNAs	**Accuracy**: 100%; Analysis time: 20 min
**65**	Optical: Colorimetric	DNA-G4 (hemin-dependent)/TMB signal-off catalytic assay	miR-155 and miR-21	**LOD: 0.62 nM (urine) / 0.43 nM (buffer)**; **Range**: 0–10 nM; analysis time: 65 min
**66**	Optical: Fluorescence	DNA nanoswitch-regulated plasmonic CRISPR/Cas12a/Au nanostars for FRET-based detection	miRNA-375 and PSA	**LOD: 17.3 fM (miRNA)** & **27.7 pg/mL (PSA)**; **Range**: 0.05–20 pM (miRNA) & 0.1–50 ng/mL (PSA); **Accuracy:** 99%; analysis time: 135 min
**67**	Optical: Fluorescence	Isothermal amplification/ hydrogel microdroplets/ Digital EXPAR	EV-derived miRNAs (let-8a, miR-223, miR-143)	**LOD: 3 fM**; **Range**: 50 fM–30 nM; **AUROC**: 0.85, 0.89, 0.92; Mix-and-read; analysis time: 50 min
OPTICAL: SURFACE-ENHANCED RAMAN SPECTROSCOPY (SERS)				
MicroRNAs				
**20**	Optical: SERS	Electroactivated nanoplatform	Exosomal miRNAs	**LOD: 0.5 nM**; *In-situ* detection
OPTICAL: SURFACE PLASMON RESONANCE (SPR)				
MicroRNAs				
**78**	Optical: SPR	pH-responsive triplex DNA nanoswitches (TDNs) with AuNP signal enhancement	miR-183 and miR-155 (Bladder Cancer)	**LOD: 0.36 pM (miR-183)** at pH 5.0 **& 0.46 pM (miR-155)** at pH8.3; **Range**: 1 pM–10 nM
OTHER OPTICAL SENSORS				
RNAs				
**80**	Optical: dual optical microfibers	LSPR (GNR / Au bipyramid)	PSA protein / PCA3 RNA	**LOD PCA3:** $1.56{\times}10^{-14}$ M
ELECTROCHEMICAL				
RNAs & microRNAs				
**103**	Electrochemical: EIS	RNA-aptamer on AuNP-SPCE	PCA3 lncRNA	**LOD:** 1 fM in buffer; **20 fM** in artificial urine; **Range**: 0.1 pM–1 nM
**104**	Electrochemical: Voltammetry (SWV)	Thiolated ssDNA probes on Au	PCA3 lncRNA	**LOD: 1.6 ng/mL**; **Range**: 0.1–1 µg/mL; high specificity
**105**	Electrochemical: Voltammetry (SWV)	Biotinylated probes / AgNPs / streptavidin-modified SPCEs	Urinary miRNA-21	**LOD: 100 fM**; multiplexing
**106**	Electrochemical: Field-effect transistor (FET)	Miniaturized Metal-Carbide@CNT iFET / aptamer	Exosomal microRNA-122 (Liver Cancer)	**LOD: 0.12 fM**; **Range**: 1 fM–10 nM; **AUC**: 0.9776

**107**	Electrochemical: EC & ECL	Dual-mode EC/ECL (polyacrylamide - MNPs)/luminol	miRNA-183	**LOD: 4.6 (EC) /4.1 aM (ECL)**; **Range**: 0.01–1000 aM
**108**	Electrochemical	Isothermal RPA / Chronoamperometry	6-RNA Biomarker Panel	**LoQ: 100 copies**; **AUC**: 0.86–0.88; analysis time: 55 min

Table S4. Sensors and sensing systems for the detection of extracellular vehicles (EVs).

REF.	TECHNIQUE	METHOD	BIOMARKER(S)	ANALYTICAL PERFORMANCE
OPTICAL: COLORIMETRIC & FLUORESCENT				
**15**	Optical: Fluorescence	Herringbone mixers/ encoded photonic crystal microparticles/FITC	6 Exosomal Proteins	**LOD: 10^3 particles/mL**; **Range** 10^3–10^8 particles/mL; **AUC: 0.798**; Multiplex analysis
**68**	Optical: Fluorescence	Fusogenic nanoreactor/DNA probes/ toehold-mediated strand exchange/FRET	Exosomal miR-200c, miR-222, and miR-375	**LOD: 1.42×10^4, 2.95×10^3, 4.23×10^4 EVs/mL (miR-200c, miR-200c, miR-222)**; **Accuracy**: 75%; **AUROC**: 0.81; analysis time: 60 min
**72**	Optical: Colorimetric	anti-HER2 antibodies@Magnetic-Polydiacetylene nanoparticles	HER2-overexpressing EVs	**LOD: 8.5×10^8 particles/mL**; analysis time: 2 h;
**73**	Optical: Optofluidic	PDMS chip / fiber-coupled laser	Single EVs (Organoids)	analysis time: 30 s; Real-time monitoring; IoT control
OPTICAL: SURFACE-ENHANCED RAMAN SPECTROSCOPY (SERS)				
**74**	Optical: SERS Immunoassay	DNN-assisted (Au@Ag NPs)	GRB-7, CD63, HER2 EVs	Monitoring drug efficacy
**122**	Optical: SERS	Gold nanohole array for size-exclusive single EV trapping with CNN classification	Single extracellular vesicles (sEVs)	**Accuracy:** 97.37%; analysis time: 40 min; successful subtyping of urologic cancers
ELECTROCHEMICAL				
**109**	Electrochemical	Capacitance biosensor (rGO/MoS_2)	CD63 & PSMA (EVs)	**LOD: 483 EVs/µL**; analysis time: 10–30 min
**110**	Electrochemical: DPV	PCNSs/silver-carbon/MOF flexible electrodes/aptamers	EVs	**LOD: 29 particles/µL**; **Range**: 10^2–10^6 particles/µL; **Recovery**: 99–103% (Urine); **RSD**: <2.13%
**111**	Electrochemical	Dual-aptamer (MNPs/carbon nanofibers)	PSMA-positive EVs	**LOD: 3 pg/mL**; **Range**: 10–1400 pg/mL; **Recovery**: 91.8–105.4%; **RSD**: <6%

Table S5. Sensors and sensing systems for the detection of volatile organic compounds (VOCs).

REF.	TECHNIQUE	METHOD	BIOMARKER(S)	ANALYTICAL PERFORMANCE
OPTICAL: COLORIMETRIC & FLUORESCENT				
**116**	Optical: Colorimetric	Deep learning (FRR algorithm) screening of 400 electrostatic complexes of ionizable dyes/8-element array	Multi-cancer VOCs (Bladder, Colorectal, Prostate)	**AUROC**: 0.83-1.00 (BC) and 0.96 (PC); Multiclass **Accuracy**: >85%; Readout in 5 s; long incubation
**117**	Optical: Fluorescence	Sensor array of six human olfactory receptor-embedded nanodiscs	Prostate cancer VOCs	**AUROC:** 0.96; **Accuracy:** 0.890; **Precision:** 0.928; **Recall:** 0.856; **F1-score:** 0.889; **Specificity;** 0.927
ELECTROCHEMICAL				
**112**	Electrochemical: eNose	Metal oxide gas sensor array with SPME headspace sampling and RBF neural network	Urinary VOCs	**AUC**: 0.88; **Specificity**: 0.99; **Sensitivity**: 0.44
**124**	Electrochemical: eNose & eTongue	Data fusion / AI algorithms	VOCs and non-volatiles	**Accuracy:** 97.3%; **Specificity:** 97.8% (data fusion of eNose & eTongue)
**125**	Electrochemical: eNose & eTongue	SVM/KNN analysis	VOCs and Zinc	**Accuracy:** 92.9% (eTongue) / 100% (eNose)
**126**	Electrochemical	Potentiometric eTongue	Urinary ionic composition/VOCs	**Accuracy: 77-87%**; ML-assisted; analysis time: 2 min

Table S6. Sensors and sensing systems for the detection of RNA nucleosides & cancer cells.

REF.	TECHNIQUE	METHOD	BIOMARKER(S)	ANALYTICAL PERFORMANCE
OPTICAL: COLORIMETRIC & FLUORESCENT				
RNA NUCLEOSIDES				
**56**	Optical: Chemiluminescence	Aptamer-sensing and Au@ZIF67 catalysis combined with target cycle amplification and luminol as label	Adenosine	**LOD: 1.5 pM**; **Range**: 10 pM–100 nM; **Recovery**: 97.6–101.7%; analysis time: 90 min
**61**	Optical: Fluorescence	Aptamer strand displacement for FRET-based turn-on sensing	N^1-methyladenosine	**LOD: 1.9 μM**; **Range**: 0-25 μM; **Recovery:** 86–116%; analysis time: 30 min
**62**	Optical: Fluorescence	Tetralactam macrocycle-based array / SVM-regression analysis for ternary mixtures	16 RNA Nucleosides (Breast, colorectal & urogenital cancer)	**LOD: 1.1 μM (adenosine)**; **Range:** 0–60 μM; **Accuracy**: 100%; analysis time: 20-30 min
CANCER CELLS				
**115**	Optical/ Microfluidic Flow Cytometry	Machine learning (SVM) assisted analysis	Exfoliated bladder cancer cells	**Accuracy:** 84.7%; **Specificity:** 91.0%; **Sensitivity:** 75.0%; analysis time: 15 min
PHOTOTHERMAL				
CANCER CELLS				
**114**	Photothermal	MnO_2@CuS nanoprobes	HT-1376 Cells (Bladder cancer)	**LOD: 2 cells/mL**; **Range**: 10–10^6 cells/mL; **Recovery**: 98.2–102.0; **RSD**: 1.7–3.2%

* Lowest LODs are highlighted in red
